# Synergistic effects of abietic acid combined with doxorubicin on apoptosis induction in a human colorectal cancer cell line

**DOI:** 10.1038/s41598-025-99616-2

**Published:** 2025-05-08

**Authors:** Hesham Haffez, Hend H. Sanad, Hassan Ebrahim, Zeineb A. Hassan

**Affiliations:** 1 Biochemistry and Molecular Biology Department, Faculty of Pharmacy, Helwan University, Cairo, 11795 Egypt; 2 Center of Scientific Excellence “Helwan Structural Biology Research, (HSBR)”, Helwan University, Cairo, 11795 Egypt; 3 Health Affairs Directorate, Mansoura Health Administration, Mansura city, , El Dakahlia Egypt; 4 Pharmacognosy Department, Faculty of Pharmacy, Helwan University, P.O. Box 11795, Cairo, Egypt

**Keywords:** Abietic acid, Doxorubicin, Combination therapy, Colorectal cancer, Toxicity, Drug development, Biochemistry, Cancer

## Abstract

Cancer is a significant global disease with high mortality and limited therapeutic options. Chemotherapy is a cancer treatment option; however, there are still issues, including severe side effects, inadequate response, and drug resistance. Abietic acid is a natural diterpene with diverse pharmacological properties and can be used for cancer treatment. Therefore, this study aimed to assess the anticancer efficacy of abietic acid in combination with doxorubicin, a highly clinically used chemotherapeutic agent. Biochemical investigations include initial viability assays, combination therapy using isobologram analysis, apoptosis and cell cycle assays, gene expression assay, ELISA analysis of protein expression, DNA fragmentation, and wound healing assays. The data showed that doxorubicin-abietic acid (DOX-AB) is an effective and safe anticancer combination for Caco-2 cells. DOX-AB had a high safety index with minimal cytotoxicity at the combination dose on normal WI-38 fibroblasts cells. DOX-AB significantly decreased the proliferation and viability of Caco-2 cells, with an increase in the apoptosis rate in the late stage and necrosis with cell cycle arrest at the G_2_/M phase. Significant changes in the expression of modulators related to apoptosis, inflammation, and epigenetics were observed in gene and protein levels. DOX-AB combination had more efficient anticancer activity than doxorubicin alone. This study suggested that the use of abietic acid in combination with doxorubicin is a promising treatment for colorectal cancer because it enhances doxorubicin activity at relatively low doses with minimal cytotoxicity and overcomes multidrug resistance in tumors; these findings merit further investigation.

**Supplementary Information:**

The online version contains supplementary material available at 10.1038/s41598-025-99616-2.

## Background

Cancer is a major global health concern, causing increasing incidence and mortality rates^1^. Treatment options include surgery, radiation, chemotherapy^2^, and immunotherapy^3^. However, chemotherapeutic resistance^4–6^and significant side effects have increased, reducing their efficacy^7–9^. Therefore, there is a usual need for effective treatment strategies for management of different malignancies. Example for anticancer agents is doxorubicin that was extracted from *Streptomyces peucetius*in the late 1960 s^8,10,11^, and have shown promising antitumor effects on most malignancies^10,11^. However, its high hydrophilicity, short half-life, low bioavailability, and high volume of distribution compromise its effectiveness^11,12^. Also, the high doses of doxorubicin can lead to adverse effects like cardiotoxicity, extravasation, nephrotoxicity, and myelosuppression^11,12^. Doxorubicin resistance is also known due to tumor characteristics, apoptotic process disturbance, autophagy suppression, enzyme patterns changes, gene mutations, and drug efflux, which diminish its effectiveness^12–15^. Another example is 5-Fluorouracil (5-FU) that was discovered fifty years ago, and widely used chemotherapeutic agent for treating common cancers like skin, brain, breast, and colon cancers^16,17^. However, its efficacy is limited in colorectal cancer^18^due to fast metabolism, short half-life, minimal bioavailability, lethal effect, and insufficient selectivity for tumorous cells^19^. A third new synthetic derivative is Raptinal, triggers the caspase-dependent intrinsic apoptosis pathway by activating caspase-3^20^, causing cell death and selective anticancer activity in hepatocellular carcinoma animal model^21,22^. It also triggers p53 expression and reactivates its mutant form, making it a promising approach for anticancer therapies, especially for challenging-to-cure cancers^21^. Raptinal treatment induces aggressive apoptotic effect that may cause side effects such as renal and cardiac toxicities^22^. Consequently, it was essential to identify less toxic and inexpensive systematic therapy procedures that are also more efficient.

Natural products (NPs) are crucial in the development of new therapeutic discoveries, with approximately 60% of anticancer medications used in clinical settings being direct, derived, or inspired by NPs^23–25^. Tumor therapy heavily relies on natural substances like paclitaxel and camptothecin derivatives^26–28^. Phytochemicals like curcumin and quercetin are important for enhancing patient survival when used in conjunction with antitumor or combination therapies^28–32^. Abietic acid (AB) is an example of a natural compound derived from *Pinus*species, and has diverse pharmacological activities, including antiobesity, antiallergenic, anticonvulsant, and anti-inflammatory properties^33^. Studies have shown that AB protects against cancer by reducing the expression of oncogenic genes like VEGF, TGF-β, and NF-κB in non-small cell lung and breast cancer^34–36^. A recent study found that AB has therapeutic effects on cancer cells by inducing apoptosis and cell cycle arrest and reducing the expression of proliferation genes like c-myc, TNF-α, NF-κB, VEGF, TGF-β1, and IGF1R, which are known for their potential effects on angiogenesis, proliferation, metastasis, and invasion^42,43^. Other studies have shown successful antitumor properties of natural metabolites like Silymarin, Luteolin and Quercetin when combined with anticancer drugs as doxorubicin^37–39^. Also, successful combination of Diosmetin, Zerumbone, Thymoquinone and Melatonin with 5-FU^40–44^. However, few studies have investigated their detailed mechanistic effects^45–47^.

**Fig. 1:**
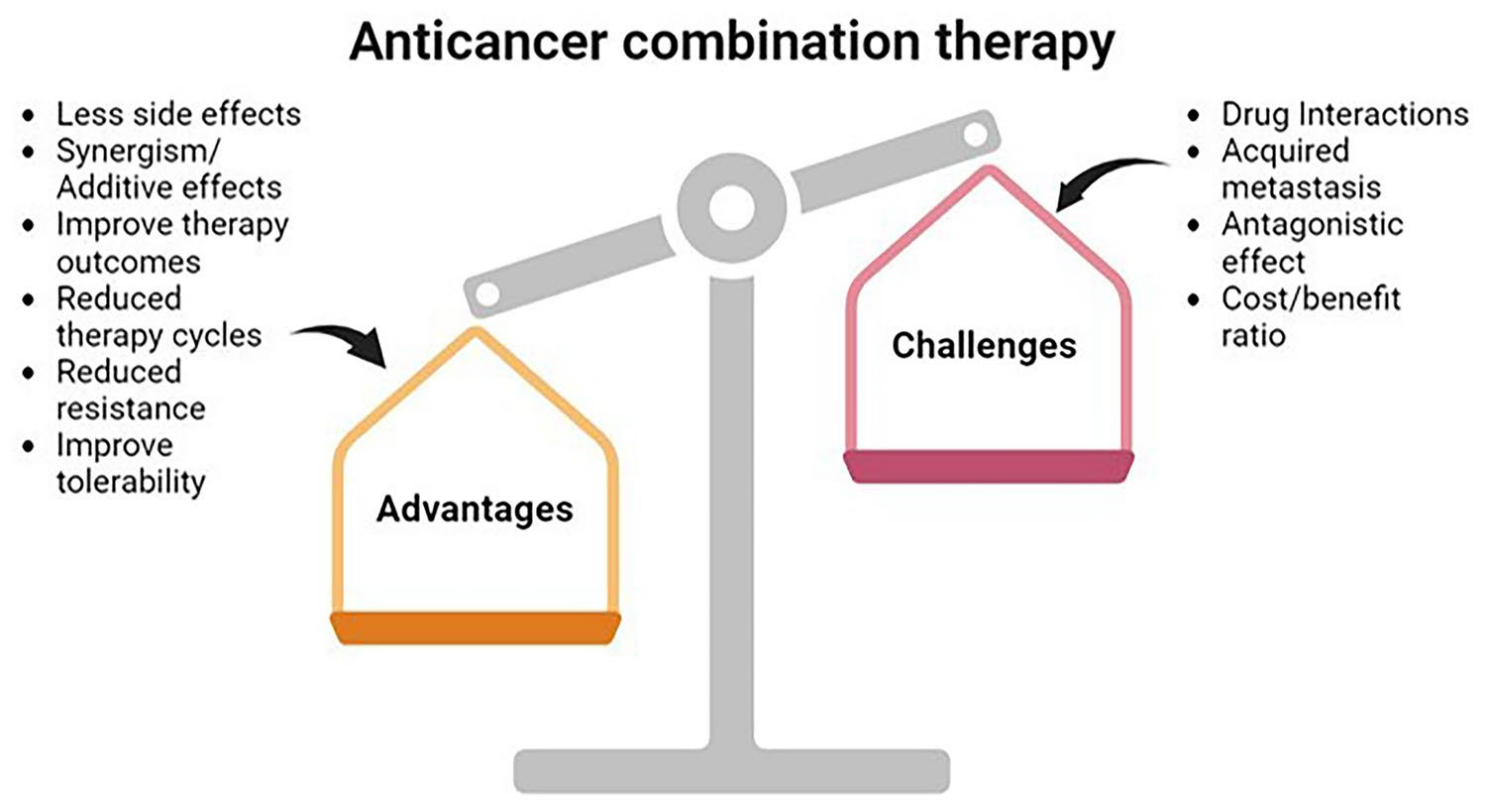
The advantages and challenges of using anticancer combinatorial approach.

Combination therapy is a popular approach in cancer treatment due to its numerous benefits. It enhances treatment outcomes by producing better therapeutic benefits and synergistic anticancer activity^2^. Combination eliminates clonal heterogeneity, leading to higher response rates^48^. It allows for the use of individual medications at lower doses while maintaining therapeutic efficiency, reducing toxicity^49^. Combination of medications act by distinct methods, reducing the likelihood of resistant cancer cells arising^2^. They also selectively eliminate cancer stem cells, reducing drug resistance and reducing the likelihood of cancer relapse^50^. Combination therapy may be beneficial for advanced tumors that do not respond to conventional treatments like radiation or surgery^51^. Combination treatment eliminates cellular processes linked to adaptive resistance and simultaneously attacks multiple molecular pathways necessary for cancer cell survival^52^. These benefits and challenges can be summarized in Fig. 1. Therefore, this is study aims to assess the effect of the combination of abietic acid and anticancer drugs in selected cancer cell lines through initial screening. Additionally, this study aims to understand the underlying anticancer mechanisms of the combination effect compared to those of a single drug, which might aid in the decision-making process for potential therapeutic applications.

## Methods

### Cell culture and materials

The tested cancer cell lines include human liver cancer cell line (HepG2), epidermoid carcinoma of the larynx cell line (Hep-2), breast cancer cell line (MDA-MB231), human colon cancer cell lines (HCT-116 and Caco-2) and prostate cancer cell lines (PC-3 and DU-145). Fibroblast lung cells (WI-38) were used as a normal cell line for comparison and calculation of the selectivity index. All cancer cell lines, along with normal cells were retrieved from the holding company’s cell culture bank for the production of vaccines, sera, and drugs (VACSERA, Giza, Egypt) with passage number 10–15. The cell lines were characterized and propagated in the Center of Scientific Excellence “Helwan Structural Biology Research, (HSBR)” in accordance with the standard protocol^53–57^. These cell lines were cultured under standard conditions with 10% FCS, 2 mM M-glutamine, and 100 IU penicillin/streptomycin. The standard culture conditions included growth at 37 °C and 5% CO_2_. After 3–4 days, the culture medium was changed every 2 days to 85–90% confluence to facilitate rapid cell division. For use in downstream applications, cell cultures were passage through a 0.25% trypsin/EDTA solution. Abietic acid was purified from *Pinus palustris* Mill. (*Pinaceae*) tree and characterized for percent purity, which was calculated in correlation with the percent area under each peak and was found to be 98.46% by HPLC according to the standard protocol^55^. Stock solutions of the drugs (doxorubicin and 5-FU) were prepared using DMSO (Sigma‒Aldrich, St. Louis, MO, USA) to a final concentration of 10 mM. The stock solutions were then divided into aliquots and kept at −20 °C until further use. All materials were preserved in compliance with the guidelines provided by the manufacturer.

### Viability assay

The 3-(4,5-dimethylthiazol-2-yl)−2,5-diphenyltetrazolium bromide (MTT, Serva) colorimetric test was used to evaluate antiproliferation and cytotoxicity according to a previously used protocol^53,55,58,59^ Five dilutions (0.01, 0.1, 1, 10, and 100 µM) of doxorubicin, 5-FU and abietic acid were used for treatment for 24 h. Cells treated with 0.1% DMSO alone served as the negative control group. A microplate reader (800 TSUV Biotek ELISA Reader) was used to measure the amount of soluble formazan generated at 570 nm. The concentration of drug that inhibited 50% of the cell growth was determined as the IC_50_using a 4-parameter logistic nonlinear model^60,61^. The safety index (SI) was calculated using WI-38 normal human fibroblasts as the IC_50_ drug (WI-38)/IC_50_drug (cancer cell line) ratio^62^. The efficiency and safety of anticancer drugs are indicated by lower IC_50_values and SI values greater than 1^63^.

### Isobologram analysis of the combined drug effect

The anticancer effects of the standard chemical drugs doxorubicin and 5-FU in combination with abietic acid or Raptinal on Caco-2 cancer cells were assessed separately using the isobologram method to enhance the efficacy of these drugs at reduced toxic IC_50_doses^53,64,65^. A set ratio of combination drugs was applied to cells concurrently at dosages that generally corresponded to 4, 2, 1, 0.5, 0.25 and 0.125x the individual IC_50_values of each drug and abietic acid. Multiple-drug effect analysis was first performed by Chou and Talalay at a 1:1 mixture ratio^66,67^. The combination index (CI) can be calculated using the following mathematical Eq. (1)^68^:

1 $$\text{CI} = \text{d1/D1} + \text{d2/D2}$$

The dosages of drugs 1 and 2 that, when administered together, elicit a certain reaction are designated d1 and d2. The dosages of drugs D1 and D2, when administered separately, result in the same reaction. Moreover, antagonism was defined as CI > 1, additivity as CI = 1, and synergy as CI < 1.

Equation (2) is used to determine the DRI, which is defined as “the fold decrease in the dose of each drug if two drugs are given in combination, as opposed to individual treatment, to achieve a particular level of cytotoxicity^69^.

2 $$\text{DRI (for drug 1)} = \text{D1/d1}$$

A higher DRI indicates a more advantageous drug combination since a DRI > 1 indicates a beneficial dose decrease. A DRI less than one suggests an inefficient combination and an adverse dosage reduction. A DRI of 1 indicates that the dosage of the chosen medication was not lowered^70^. The same previously mentioned protocol was performed using HCT-116 resistant colorectal cancer cell line at determined IC_50_ doses of Doxorubicin and Abietic acid.

### Morphological assessment of Caco-2 cells treated with doxorubicin, abietic acid and doxorubicin combined with abietic acid

Morphological and nuclear alterations were observed under an Axio Observer 7 inverted phase contrast fluorescence microscope at 40× magnification after treatment with single drugs or their combination for 24 h. Caco-2 cells treated with 0.1% DMSO were used as a negative control.

### Apoptosis assay using Annexin V (AV)/propidium iodide (PI)

The Annexin V (AV)/Propidium Iodide (PI) assay is useful for assessing the apoptotic effect in many different cancer cell lines in accordance with the detection kit’s procedure (Beckman Coulter, Brea, CA, USA city, state abbreviation for USA, nation) and our standard protocol^53–55^. Briefly, the cell suspension was cultivated in 25 cm^2^ flasks for 24 h until more than 90% confluency. Caco-2 cells were seeded in 6-well culture plates (1.5 × 105 cells/well) in 2000 µl culture medium for the next 24 h. Subsequently, the cells were treated with the IC_50_ dose of the single drugs or the combined IC_50_for another 24 h. After being washed with phosphate-buffered saline (PBS) (Lonza)., the cells were resuspended in 1 mL of binding buffer. 100 µL of cell suspension was treated with 1 µL of FITC-labelled Annexin-V and 5 µL of PI and incubated for 15 min at 4 °C in the dark. Then, 400 µL of ice-cold binding buffer was added to the suspension, and each sample’s apoptotic cells were examined using a Beckman Coulter Epics XL flow cytometer. The amount of apoptotic cell death reported as a proportion of all gated cells was used to determine the apoptotic index (AI)^71,72^. Apoptosis analysis was performed using Beckman Coulter CytExpert software (version 2.4.0.28). The same previously mentioned protocol was performed using HCT-116 resistant colorectal cancer cell line at determined IC_50_ doses of Doxorubicin and Abietic acid.

### Cell cycle analysis

Cell cycle analysis was performed according to a standard protocol^54,55^, as a total of 1 × 10^6^Caco-2 cells were seeded into a 25 cm^2^ flask for 24 h until they reached 90% confluence. Cells were split and seeded in 6-well plates and treated with the IC_50_ of single drugs or a combination of drugs for 24 h. The cells were collected, preserved for an overnight period in 70% alcohol, and then stained with PI (50 µg/mL). The Cytoflex flow cytometer (Beckman Coulter, USA) was used to quantify the DNA content. The cell cycle analysis with G_0_-G_1_, S, and G_2_M cells were gated using Beckman Coulter CytExpert software (version 2.4.0.28). The same previously mentioned protocol was performed using HCT-116 resistant colorectal cancer cell line at determined IC_50_ doses of Doxorubicin and Abietic acid.

### Gene expression analysis of several key regulatory genes

Gene expression analysis of some regulatory genes related to apoptosis, inflammation and epigenetics was conducted using Caco-2 cells. After the cells were seeded in 6-well plates in triplicate at a density of 1 × 10^6^ cells per well under standard incubation conditions, the cells were treated for 24 h with IC_50_ doses of single and combination therapies in addition to the solvent control (0.1% DMSO)-treated cells. The cells were extracted, and a Favour-PrepTM Blood/Cultured Cell Total RNA Purification Micro Kit (Favorgen Biotech Corp., Ping-Tung, Taiwan) was used to purify the total RNA. RNA was reverse transcribed into first-strand cDNA using the Revert Aid First Strand cDNA Synthesis Kit (Thermo Scientific, Waltham, MA, USA). The kit for qPCR utilizing HERAPLUS SYBR^®^ Green (Willowfort, Nottingham, UK) was used for the amplification of genes. Gene expression was analysed and quantified using the 2ˆ^−ΔΔCT^method^73^. The sequences of the primers used are listed in Table **S1**. The fold change in Bax and Bcl-2 genes was assessed, and the Bax/Bcl-2 ratio can serve as a prognostic marker that indicates whether a cell will undergo apoptosis^74–76^. A lower value for this ratio indicates that human cancer cells may be resistant to apoptosis^77,78^.

### ELISA assay of key apoptotic proteins expression activity

Cancer cells were treated with IC_50_ doses of abietic acid, doxorubicin and their combination in comparison to 0.1% DMSO as negative control for 24 h in order to assess Cyt-C, Casp-3, p53, Bax and Bcl-2 proteins expression in cell lysates. The cell pellets were collected by centrifugation and the supernatant were removed and pellets were washed 3 times with PBS followed by resuspension in 500µL RIPA lyse buffer. Following the manufacturer’s instructions, commercially available ELISA kits (Cyt-C- Cat. No. MCTC0, Casp-3- Cat. No. E-EL-M0238, p53- Cat. No. MBS2610658, Bax- Cat. No. LS-F21313 and Bcl-2- Cat. No. E0778 m) were used to estimate the total proteins expression using standard curve calibration. ELISA plates were coated with the specific capture antibody, and 5% BSA in PBS-Tween solution was used to block the coating for an hour. After two hours of incubation, a secondary HRP-detection antibody was added, and the samples were left to incubate for a further two hours followed by measuring the colorimetric absorbance of samples, standards, control and blank at 450–570 nm.

### DNA fragmentation assay

One characteristic feature of apoptosis is DNA fragmentation, and a colorimetric diphenylamine test was used to quantify DNA fragmentation in Caco-2 cells according to our standard protocol^54,79–81^. Briefly, 1 × 10^6^ cells were grown to confluency for 24 h. Cells were treated with single drugs at the IC_50_ or in combination for 24 h. Then, the cells were carefully scraped off and pelleted using a 300x g centrifuge at 4 °C for 10 min. The cells were resuspended in 0.8 ml of 0.01 M PBS (pH 7.4), 0.7 ml of ice-cold lysis buffer (pH 8.0), 0.5% Triton X-100, 20 mM EDTA, and 5 mM Tris for lysis. The mixture was incubated for 15 min at 4 °C to guarantee full lysis. The cell lysate was centrifuged at 13,000 × g and 4 °C. DNA fragments are now found in the supernatant, whereas whole DNA is found in the pellets. After the supernatant was poured into a 5-ml glass tube, 1.5 ml of 10% trichloroacetic acid (TCA) was added, and the mixture was allowed to sit at room temperature for 10 min. Both intact and fragmented DNA were extracted using centrifugation at 500x g for 15 min at 4 °C, followed by boiling at 100 °C to release the inorganic phosphate. The pellet containing DNA was again suspended in 0.7 millilitres of 5% TCA and allowed to cool to room temperature. After centrifuging the suspensions at 300 × g for 4 °C, 0.5 ml of the supernatant was transferred to a fresh glass tube and incubated overnight at 30 °C. The color was finally produced using 1.5 g of diphenylamine mixed in 100 ml of acetic acid and 1.5 ml of H_2_SO_4_with acetaldehyde at a final concentration of 16 mg/mL. The absorbance intensity of both the supernatant and the sample was measured colorimetrically at 600 nm. The relative ratio of low-molecular-weight fragmented DNA to the sample’s total DNA content was used to represent DNA fragmentation^81^.

### Wound healing assay

A total of 1.5 × 10^5^ Caco-2 cancer cells were seeded in 6-well plates with 2000 µl of culture media and incubated for 24 h. Once the cells reached more than 90% confluency, the cancer cell monolayers in each well were scraped with a sterile pipette tip. Next, the cells were treated with the single drugs at the IC_50_ or in combination and were incubated for 24–48 h. Finally, the cell migration rate was calculated as the percentage of wound closure or area decrease according to Eq. (3)^82^:

3 $$\text{Wound Closure}\: {\%} = \text{[A}_0-\text{A}_\text{h}/\text{A}_0]\: \text{X}\: 100$$

A_0_ is the area of the wound measured immediately after scratching (t = 0 h).

A_h_ is the area of the wound measured hours after the scratch was made.

The calculation of the scrap width was performed using an Axio observer 7 inverted phase contrast fluorescence microscope, and the calculations were performed using Zen 3.8 software.

### Histone deacetylases (HDAC) activity/inhibition direct assay

Histone deacetylases (HDACs) catalyse the hydrolytic removal of acetyl groups from histone lysine residues, which is a crucial step in the transcriptional regulation of gene expression in eukaryotic cells. Human cancer formation, cell proliferation, and cell cycle regulation are all closely correlated with HDACs. Hence, it was essential to understand the effect of single drugs and combination on total cellular HDAC activity. Total HDAC activity/inhibition can be measured using the HDAC Activity/Inhibition Assay Kit (Cat. No. KA 0628, Abnova, USA). Trichostatin (TSA) at 1 µM served as the positive control^83^. The special acetylated histone substrate is steadily collected on the strip wells in an experiment using this kit. Histone substrate is bound by active HDACs, which then deacetylate it and the residual un-deacetylated substrate can be identified by high affinity acetylated histone antibody and developed color measured colorimetry^84–87^. The quantity of the un-deacetylated histone can be measured using standard curve, which is inversely proportional to HDAC enzyme activity. Briefly, 1 × 10^6^ Caco-2 cells were grown for confluency for 24 h. Cells were treated with IC_50_ doses of single drugs and combination for 24 h in 6-well plates. Cells were lysed and nuclear extracts were separated according to the standard protocol. To prepare the standard curve, in provided 96-wells strips, 50 µL of diluted wash buffer was added (without addition of Biotinylated HDAC substrate). Then, 1 µL of HDAC Assay Standard (20 µg/mL) was used in varying amounts (0.1–10 ng), and the mixture incubated at room temperature for 45 min. For preparation of samples, 50 µL of Biotinylated HDAC substrate were combined with 50 µL of HDAC assay buffer followed by addition of 2 µL nuclear extract corresponding to each sample and the mixture was incubated for 60 min at 37 °C. After the incubation period ended, each well received 50µL of capture antibody, and the plate was incubated for 60 min at room temperature. 50 µL of detection antibody were added to each well and incubated at room temperature for 30 min. 100 µL of developing solution were added to each well and incubated at room temperature for 30 min. To terminate the enzymatic process in the standard wells with greater concentrations of standard control, 50 µL of stop solution was added to each well when the color turned medium blue. The color should become yellow within 5 min and the absorbance should be readable at 450 nm on a microplate reader. The total percentage of HDACs inhibitory activity was calculated according to the following Eq. (4):

4 $$\begin{aligned} \text{Inhibition}\:{\%}\: =&\: \text{(1- [OD (positive control-Blank)-OD (sample-Blank-)]/ OD (positive control-}\\&\quad\!\text{Blank)-OD (negative control-Blank)]) X} \:100 \end{aligned}$$

### Statistical analysis

The data are expressed as the means ± SEMs. The statistical software GraphPad Prism 8.0.2 (CA, USA) was used for data analysis. One-way ANOVA of variance with repeated measures was used to assess group means. This was followed by post hoc Dunn’s test and Tukey’s multiple comparisons test for post hoc assessment of individual means. *P* < 0.05 was considered to indicate statistical significance.

## Results

### Cancer cell viability

MTT assay was used to assess various tested compounds, including two known chemotherapeutic drugs (Doxorubicin and 5-FU), one synthetic compound (Raptinal) and one natural compound (abietic acid), at different concentrations (0.1–100 µM) for 24 h in different tested cancer cell lines, and the results were compared to those in a normal cell line (WI-38). Interestingly, all of the compounds displayed antiproliferative activity in a dose-dependent manner, with IC_50_ values ranging from 8.16 to 31.56 µM for 5-FU, 11.22–35.35 µM for doxorubicin, 13.73–51.11 µM for abietic acid and 6.78–24.19 µM for Raptinal, as shown in Table 1; Fig. 1S. Regarding the safety index, 5-FU showed the minimal cytotoxicity to normal cells, with an SI ranging from 2.8 to 10.8, followed by doxorubicin (0.9–2.8) with IC_50_ value of 35.35 µM on MDA-MB231 higher than its IC_50_ on WI-38 normal cells. Abietic acid, a natural compound, showed a similar safety index to chemotherapeutic drugs (1.8–6.5), followed by the lowest Raptinal (0.24–0.9), as shown in Table 1; Fig. 1S. Raptinal showed major IC_50_ values on cancer cell lines higher than its IC_50_ values on WI-38 normal cells followed by doxorubicin. This may suggest that all the tested compounds have antiproliferative effects on various types of cancer cells. However, Raptinal followed by doxorubicin showed low safety, suggesting that it has serious side effects and toxicity. The higher IC_50_values observed for Raptinal and doxorubicin in the cancer cell lines compared to the normal WI-38 cell line may be explained by several factors. Cancer cells often exhibit heterogeneous responses due to altered metabolic pathways, genetic mutations, overexpression of drug efflux pumps, or enhanced repair mechanisms, which can contribute to their resistance to certain compounds^88,89^. Also, the compounds may have a higher baseline toxicity in normal cells due to differences in cellular metabolism, proliferation rates, or the presence of specific pathways that make them more susceptible to the compounds^90^, especially Raptinal that is known for its rapid induction of apoptosis^20,21,91,92^. Also, normal cells might rely more heavily on the targeted pathway for survival, making them more sensitive to inhibition^93^. Finally, these chemical compounds might interact with off-target proteins or pathways that are more critical in normal cells, leading to increased toxicity in WI-38 cells compared to certain cancer cell lines^94^. On the other side, abietic acid as natural compound has moderate antiproliferative activity with considerably lower expected toxicity to normal cells in anticancer applications. This data may be interesting for potential further assessment for a combination between natural product with safe margin with slightly toxic anticancer agents for future clinical applications. Colorectal cancer is among the top three cancer types in terms of incidence and ranks second in terms of death, with an estimated ~ 9.5 million deaths annually^95,96^. Our earlier research has shown that Caco-2 cells of the colorectal cancer (CRC) type are among the most sensitive cancer cell lines for drug screening in-vitro^53^. This particular disease type was chosen for a number of reasons, including the global need for novel treatments for this sort of cancer given the clear dearth of FDA-approved treatments and the apparent lack of CRC cures^95^. CRC is one of the cancer types that is most frequently diagnosed in both men and women, and it is the third most common cause of cancer-related deaths worldwide^97^. Furthermore, a great deal of drugs approved for the treatment of colorectal cancer (CRC) are monoclonal anti-bodies, which are more costly than using natural products or already used drug on the market such as doxorubicin or 5-FU. Furthermore, using available anticancer medications to treat CRC is linked to increased cytotoxicity in comparison to normal cells^98,99^. Finaly, carcinogenesis, metastasis, resistance to treatment, and the formation of cancer from normal colon mucosa are all associated with specific alterations to different cell signaling pathways in Caco-2. It is also a well-characterized in-vitro cell culture system that is highly reliable for detecting multidrug resistance. For these reasons, it was important to use Caco-2 cell line for further investigations and to understand the molecular mechanism behind the antiproliferative activity and efficacy of abietic acid and standard chemotherapeutic agents in Caco-2 cells.

**Table 1:** MTT assay of two chemotherapeutic agents (doxorubicin and 5-FU) compared to a natural compound (abietic acid) and a synthetic compound (Raptinal) using a panel of various cancer cell lines and normal fibroblasts (WI-38). All the antiproliferative activities of all the compounds were measured as the IC_50_ (µM). The safety index (SI) was computed as the IC_50_ of the compound (WI-38)/IC_50_ of the compound (cancer cell line). The IC_50_ is displayed as the mean ± sem; *n* = 3.

Measured IC_50_ (µM)								
Cell lines	5-FU		Doxorubicin		Abietic acid		Raptinal	
	IC_50_	SI	IC_50_	SI	IC_50_	SI	IC_50_	SI
WI-38	88.0 ± 5.4		31.73 ± 3.9		89.75 ± 6.4		6.0 ± 1.1	
PC-3	31.56 ± 6.2	2.8	22.49 ± 7.1	1.4	27.61 ± 6.7	3.3	24.19 ± 7.3	0.24
DU-145	12.94 ± 3.1	6.8	26.14 ± 3.6	1.2	51.11 ± 8.4	1.8	21.29 ± 5.5	0.3
Caco-2	19.26 ± 4.2	4.6	15.20 ± 2.8	2.1	31.72 ± 6.4	2.8	7.18 ± 2.1	0.83
HepG-2	16.04 ± 2.5	5.5	11.22 ± 2.3	2.8	21.25 ± 2.4	4.2	9.92 ± 3.1	0.6
MDA-MB231	11.31 ± 3.1	7.8	35.35 ± 6.6	0.9	15.16 ± 4.1	5.9	12.29 ± 4.6	0.5
HCT-116	8.16 ± 2.4	10.8	17.53 ± 5.3	1.8	13.73 ± 1.9	6.5	8.18 ± 2.2	0.73
Hep-2	11.01 ± 3.3	8.0	19.53 ± 3.3	1.6	17.74 ± 5.7	5.1	6.78 ± 1.5	0.9

### Synergistic effect of combination drugs in Caco-2 cells

**Fig. 2:**
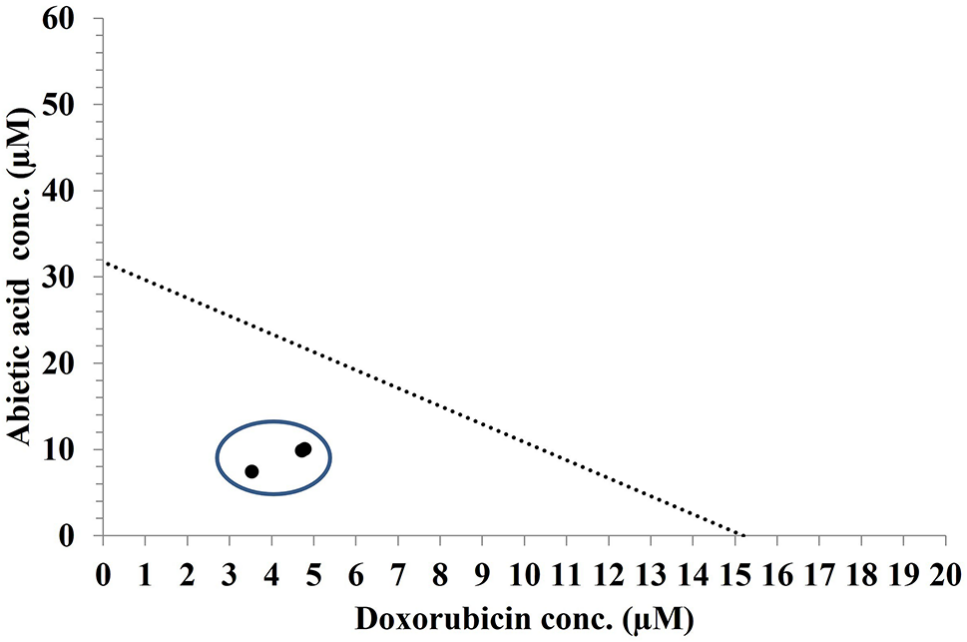
Isobologram of Caco-2 cells cotreated with abietic acid and doxorubicin for 24 h. The dotted regions that are displayed are synergistic (below). The results of the investigation showed synergism at the IC_50_ level. The means of three separate replicates are shown by the data points in circles. The data are expressed as the mean ± SEM; *n* = 3.

Using isobologram analysis, the impact of administering abietic acid or Raptinal in addition to the chemotherapeutic drugs 5-FU or doxorubicin on the Caco-2 cell line was evaluated. Four different combinations were prepared and tested on Caco-2 cells, as shown in Table 2. Using the MTT assay results as a fit for the nonlinear regression model, it was shown that the 3rd combination (abietic acid combined with doxorubicin) had the lowest IC_50_ (xIC_50_ = 0.31 ± 0.01) compared to the other combinations. This means that the combination of abietic acid with doxorubicin reduced the overall IC_50_ of the drugs by 30% of the original dose, and hence, the new IC_50_ values calculated for abietic acid in combination with doxorubicin were 10.03 µM and 4.80 µM, respectively (Table 2). These new combinatorial IC_50_ doses for abietic acid and doxorubicin were the lowest among all the other combinations (Table 2), suggesting that an exaggerated response was observed. This was confirmed by plotting the isobologram of the 3rd combination (Fig. 2), which showed that all the dose combinations were lower than the solid line in the figure plot, suggesting a synergistic effect of abietic acid with doxorubicin. Additionally, a high dose reduction index (DRI) value with a reduced combination index (CI) for the 3rd combination compared to the other combinations demonstrated a considerable reduction in the IC_50_, emphasizing the synergistic effect and substantial decrease in the doses required to achieve the same degree of antiproliferative effect (Table 2). These findings imply that the combination of abietic acid with doxorubicin, a conventional chemotherapy drug, can increase its anticancer efficacy in Caco-2 cells. To lessen the potential for large dosages to cause cytotoxicity in nearby noncancerous cells, the combined IC_50_ of abietic acid and doxorubicin was tested further on a normal fibroblast line (WI-38). Compared with the negative control, we found that the percentage growth inhibition (%GI) was 0% for abietic acid, doxorubicin or their combination while the percentage growth was 123.8% for abietic, 115.1% for doxorubicin and 112.9% for their combination (Table 2S). This likely indicates that the treatment combination has no cytotoxic effect on the normal cells. To confirm whether abietic acid-doxorubicin combination would have universal applicability to cancer cells, isobologram analysis was performed with the same protocol on the resistant HCT-116 colorectal cancer cell using pre-determined IC_50_ for abietic acid and doxorubicin single and in fraction combination. Data in Table 3S showed that xIC_50_ = 0.388 ± 0.01 suggesting synergistic effect with reduced IC_50_ doses (6.8 ± 1.6, 5.3 ± 0.9 µM for doxorubicin and abietic acid respectively). Additionally, CI = 0.63 and DRI = 4.0 suggest that combination is synergistic. This may confirm the abietic acid-doxorubicin is promising combination in colorectal cancer cell types and deserve further biochemical investigations.

**Table 2:** Combinations of individual compounds (abietic acid or Raptinal) with chemotherapeutic agents (5-FU or Doxorubicin) in Caco-2 cells using fixed ratios of the individual IC_50_ values. There was a noticeable synergistic effect when using the isobologram technique. The synergistic impact was confirmed by the combination index (CI) and dose reduction index (DRI). The data are presented as the means ± sems of *n* = 3.

Tested compounds	IC_50_ (µM)	×IC_50_ (Combination) (µM)	IC_50_ (in combination) (µM)	DRI	CI
**1** ^**st**^ ** combination**					
5-FU	19.26 ± 4.2	-------	17.62 ± 2.5	-------	-------
Abietic acid	31.72 ± 6.4	0.91 ± 0.01	29.02 ± 4.3	3.740	1.86
**2** ^**nd**^ ** combination**					
5-FU	19.26 ± 4.2	-------	54.62 ± 5.9	-------	-------
Raptinal	7.18 ± 2.1	2.8 ± 0.1	20.36 ± 3.4	5.576	15.34
**3**^**rd**^ **combination**					
Doxorubicin	15.20 ± 2.8	-------	4.80 ± 2.7	-------	-------
Abietic acid	31.72 ± 6.4	0.31 ± 0.01	10.03 ± 1.6	3.286	0.63
**4** ^**th**^ ** combination**					
Doxorubicin	15.20 ± 2.8	-------	9.87 ± 1.3	-------	-------
Raptinal	7.18 ± 2.1	0.64 ± 0.04	4.67 ± 1.0	2.446	1.30

### Changes in the morphology of Caco-2 cells treated with abietic acid, doxorubicin and their combination

**Fig. 3:**
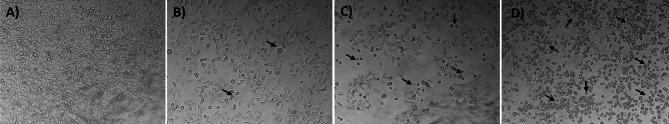
The effect of abietic acid, doxorubicin, and their combination on the growth and morphology of Caco-2 cells is demonstrated by (**A**) the 0.1% DMSO negative control, which results in a coherent and adhesive cell layer sheet; (**B**) the effect of abietic acid, which forms apoptotic colonies and separates apoptotic bodies (highlighted with a black arrow); (**C**) the effect of doxorubicin, which increases the formation of apoptotic colonies in the field (highlighted with a black arrow); and (**D**) the effect of abietic acid combined with doxorubicin, which results in more condensed and separation of apoptotic bodies (highlighted with a black arrow). The image is magnified by x40 under an inverted phase contrast microscope.

The negative control cells treated with 0.1% DMSO showed the typical morphological shape of densely packed mixed cuboidal, epithelial-like and fibroblast-like monolayer cells with some cells contain vacuoles depending on their growth stage and culture condition that were adhered to the well surface according to documented data of Caco-2 on ATCC website (Fig. 3A). When Caco-2 cells are exposed to either doxorubicin or abietic acid (Fig. 3B and Fig. 3C), cells experience moderate decreased in cell growth, cytoplasm condensation, membrane damage, loss of contact, shrinkage, and the formation of apoptotic bodies, which are indicative of apoptosis. These events can all lead to programmed cell death, including reduced cell size, fractured nuclei, and membrane blebbing. The cells treated with doxorubicin-abietic acid combination exhibited notably more morphological alterations than did the cells treated with doxorubicin and abietic acid monotherapy with high percentage of rounded apoptotic cells (Fig. 3D).

### Synergistic apoptotic effect of abietic acid combined with doxorubicin on Caco-2 cells

**Fig. 4:**
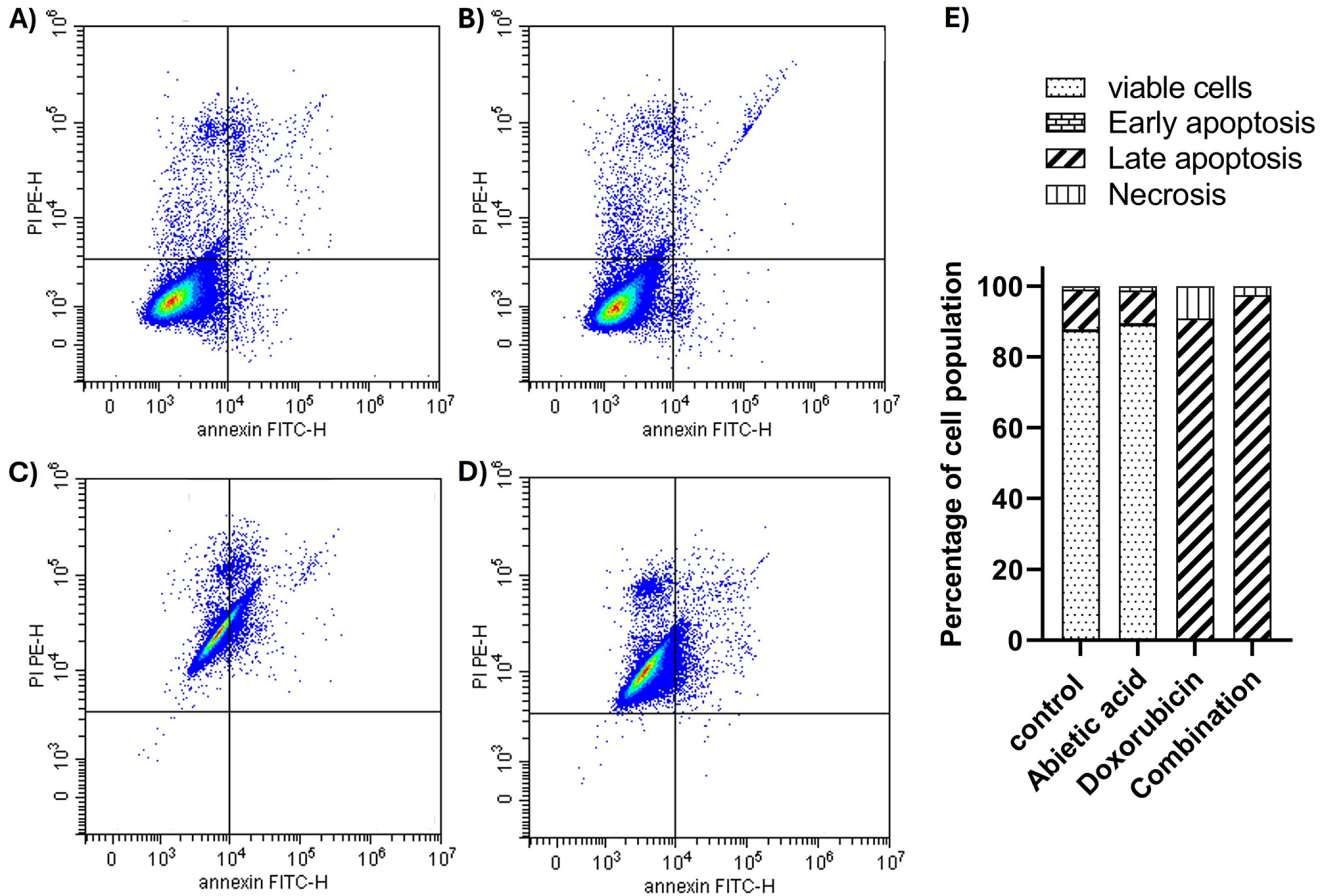
Apoptotic contour plots for caco-2 cells treated with (**A**) 0.1% DMSO act as negative control, (**B**) IC_50_ dose of abietic acid, (**C**) IC_50_ dose of doxorubicin and (**D**) combined IC_50_ doses of abietic acid and doxorubicin combination measuring the percentage of viable, early apoptotic, late apoptotic, and necrotic cells by AV/PI assay using flow cytometry. (**E**) bar graph representation illustrates the percentage of viable cells (Q1-LL), early apoptosis (Q1-LR), late apoptosis (Q1-UR), and necrosis (Q1-UL) for each treatment group. The assay was performed after the treatment of caco-2 (colon cancer) for 24 h.

It was important to understand the mechanistic effect of the combination of abietic acid and doxorubicin on Caco-2 cells using an apoptosis assay. We used flow cytometry analysis with Annexin V/PI dual labelling after treatment with the relevant IC_50_ of abietic acid and doxorubicin and their combination to examine whether the combination induces apoptosis. The negative control untreated cells showed a normal distribution, with more than 85% of the cells being viable (Fig. 4; Table 3). After treatment with either abietic acid or doxorubicin, Caco-2 cells underwent significant late apoptosis (90.81%) and necrosis (9.14%), with a reduction in viable cells to less than 1% in response to doxorubicin only, as demonstrated by Annexin V/PI dual staining (Fig. 4; Table 3). Treatment with IC_50_ of abietic acid showed a very small increase in viable cells compared to negative control that was not significant as shown in Fig. 4; Table 3. Caco-2 cells treated with abietic acid combined with doxorubicin at a reduced IC_50_ showed a significant reduction in the percentage of viable cells (< 1%) and an increase in the percentage of late apoptotic cells (97.32%) in the fraction of necrotic cells (Fig. 4; Table 3). The increase in viable cells after combination therapy is very small and not exceeding 1% that was non-significant compared to negative control. Additionally, to confirm the effectiveness of this combination on other resistant colorectal cancer cell line, HCT-116 was used for apoptosis analysis using previously determined IC_50_ combined doses in Table 1, and data showed that combination regimen was able to indue significant increase in late apoptotic and necrotic cell populations of HCT-116 with reduction in viable cells (Fig. 2S; Table 5 S) compared to negative control, abietic acid or doxorubicin. These findings imply that abietic acid and doxorubicin work together to promote apoptosis in colorectal cancer cells.

**Table 3:** Apoptosis assay analysis of the percentage of the Caco-2 cell subpopulation and distribution of cells in the various apoptosis stages as measured by flow cytometry. The IC_50_s of abietic acid, doxorubicin or their combination were applied to Caco-2 cells, and the results were compared to those obtained with 0.1% DMSO as the negative control. The combination of doxorubicin and doxorubicin dramatically reduced the percentage of viable cells through the induction of late apoptosis and necrosis. The data are expressed as the mean ± sem; *n* = 3. 2D apoptosis plot was divided into four quadrant (Lower left (LL), lower right (LR), upper left (UL), upper right (UR)).

Tested compounds	% Q1-LL	% Q2-LR	% Q3-UL	% Q4-UR
Negative control	87.46 ± 6.9	0.38	11.18 ± 2.4	0.98
Abietic	89.16 ± 8.1	0.44	9.18 ± 1.7	1.23
Doxorubicin	0.06^****####^	0.00	90.81^****####^± 5.7	9.14^**##^± 1.8
Abietic acid-doxorubicin combination	0.12^****####^	0.02	97.32^****####^± 7.8	2.55^$^± 0.1

**** indicates significance relative to the negative control at* P < *0.0001, ** indicates significance relative to the negative control at *P=*0.0069*,* ^*####*^indicates significance relative to abietic acid at *P<*0.0001*, *^##^indicates significance relative to abietic acid at* P=*0.0087*, *and *$ *indicates significance relative to doxorubicin at* P=* 0.0275*.*

### Abietic acid–doxorubicin combination induces cell cycle arrest in Caco-2 cells

**Fig. 5:**
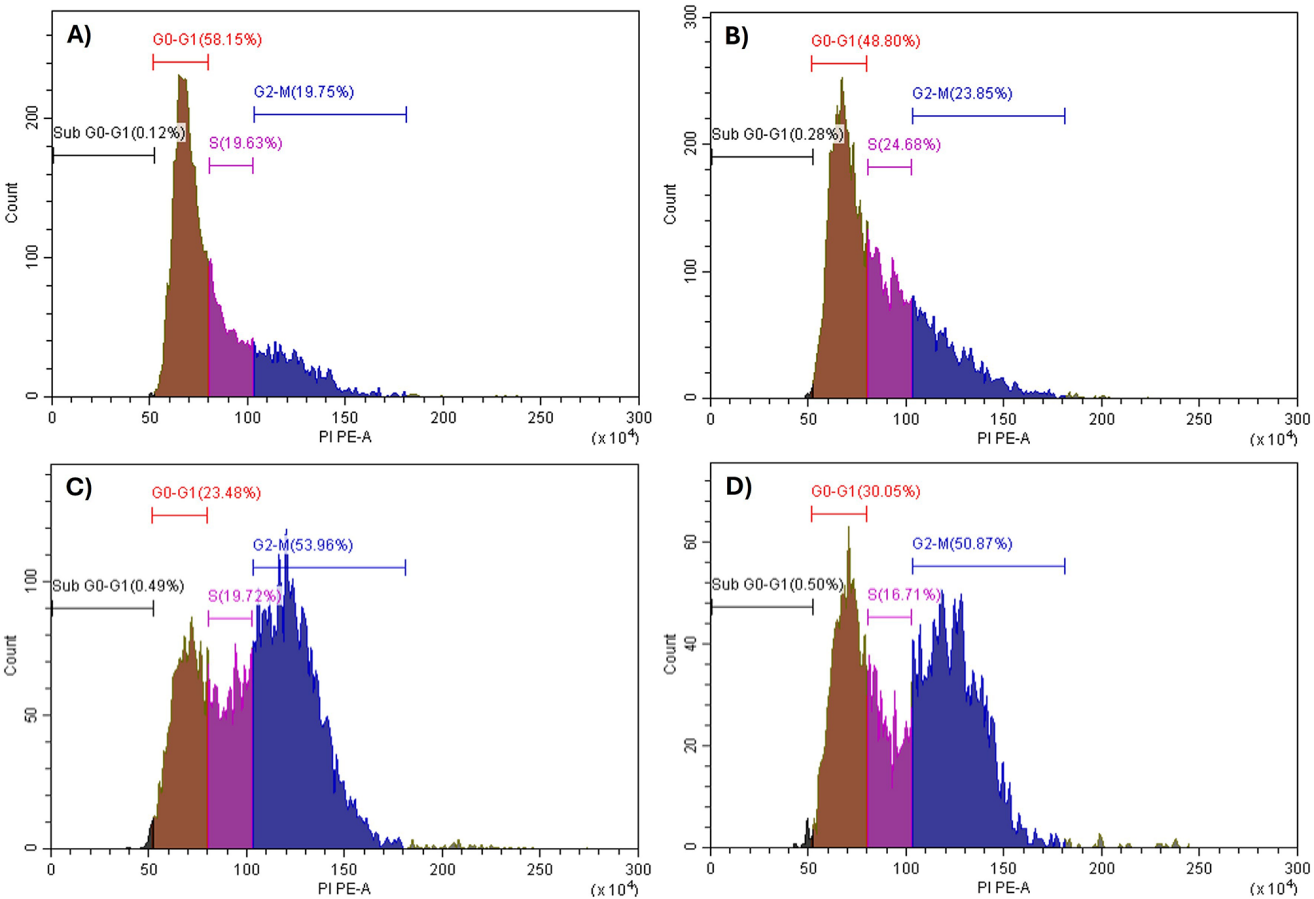
Histograms for cell cycle analysis measuring the percentage of SubG_0_-G_1_, G_0_-G_1_, S- and G_2_/M phases by PI assay using flow cytometry. The assay was performed after the treatment of caco-2 (colon cancer) for 24 h with (**A**) 0.1% DMSO act as negative control, (**B**) IC_50_ dose of abietic acid, (**C**) IC_50_ dose of doxorubicin and (**D**) combined IC_50_ doses of abietic acid and doxorubicin combination.

Doxorubicin has been demonstrated to cause cell cycle arrest in the G_2_/M phase in many cancer cell lines through DNA damage^100–102^. Since the combination of abietic acid and doxorubicin reduced cell viability, we used flow cytometry to analyse the cell cycle distribution and determine whether the combination of these two drugs enhanced cell cycle arrest in Caco-2 cells. Compared with negative control treatment, doxorubicin reduced the percentage of cells in the G_0_-G_1_ phase (28.94%) and increased the percentage of cells in the G_2_/M phase (45.93%) (Fig. 5; Table 4). The combination therapy showed a similar pattern of cell cycle arrest at relatively lower combined IC_50_ doses(Fig. 5; Table 4). Additionally, to confirm the effectiveness of this combination on other resistant colorectal cancer cell line, HCT-116 was used for cell cycle analysis using previously determined IC_50_ combined doses in Table 1, and data showed that combination regimen was able to indue significant increase in SubG_0_-G_1_ population suggesting early cell cycle arrest with significant reduction in G_0_-G_1_ phase (Fig. 3S; Table 4S) compared to negative control, abietic acid or doxorubicin. These findings imply that the combination of abietic acid and doxorubicin inhibit cell division in colorectal cancer cells.

**Table 4:** Cell cycle analysis of the percentage of the Caco-2 cell subpopulation and distribution of cells in the various cell cycle stages as measured by flow cytometry. The IC_50_s of abietic acid, doxorubicin or their combination were applied to Caco-2 cells, and the results were compared to those obtained with 0.1% DMSO as the negative control. Doxorubicin and its combination can cause dramatic cell cycle arrest at various stages of the cell cycle. The data are expressed as the mean ± sem; *n* = 3.

Tested compounds	% SubG_0_-G_1_	% G_0_-G_1_	% S	% G_2_M
Negative control	0.19	58.66 ± 6.8	19.34 ± 5.8	19.72 ± 4.1
Abietic	0.12	52.30 ± 10.1	21.69 ± 3.6	23.61 ± 5.3
Doxorubicin	0.32	28.94 ± 2.8 ^a***c###^	22.21 ± 2.6	45.93 ± 8.4 ^e***g##^
Abietic acid-doxorubicin combination	0.26	35.88 ± 5.9 ^b**d#^	17.41 ± 3.5	44.46 ± 9.5 ^f***h##^

^a***^indicates significance relative to the negative control at* P* = 0.0001, ^b**^indicates significance relative to the negative control at *P* = 0.0011, ^c###^indicates significance relative to abietic acid at *P* = 0.0009, ^d#^indicates significance relative to abietic acid at *P* = 0.0130, ^e***^indicates significance relative to the negative control at *P* = 0.0005, ^f***^indicates significance relative to the negative control at *P* = 0.0009, ^g##^indicates significance relative to abietic acid at *P* = 0.0020, and ^h##^indicates significance relative to abietic acid at *P* = 0.0035.

### Gene expression analysis of genes related to apoptosis and inflammation after treatment with abietic acid-doxorubicin combination therapy in Caco-2 cells

**Fig. 6:**
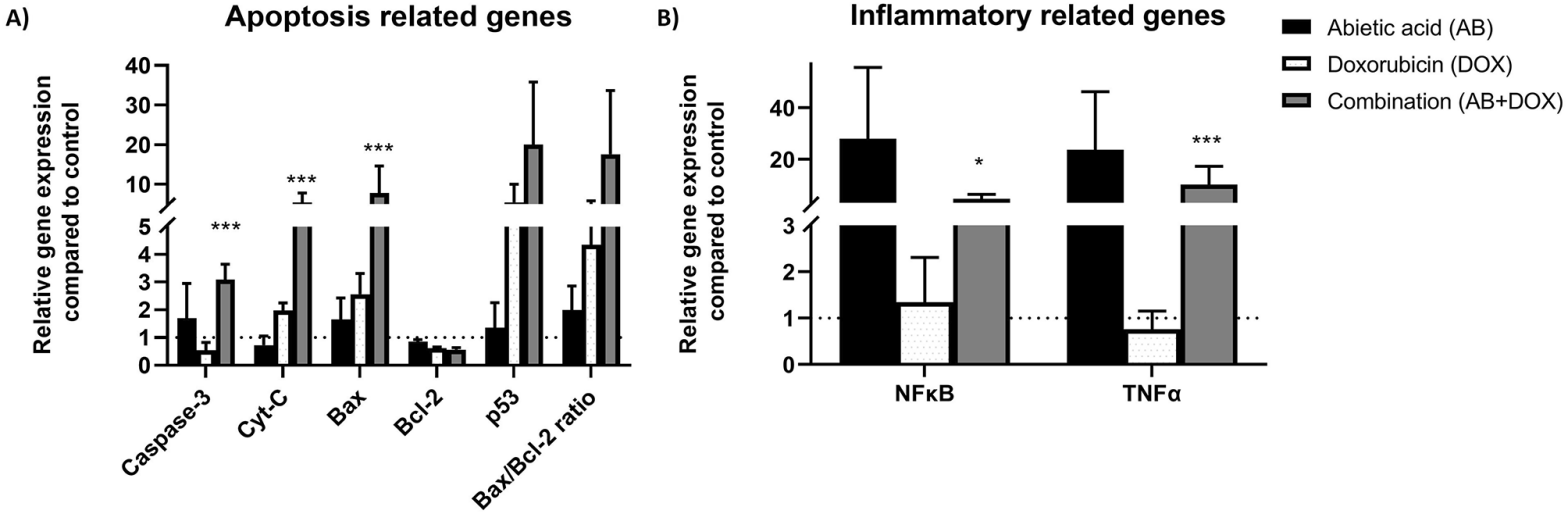
The effects of abietic acid, doxorubicin and their combination in Caco-2 cells on the expression of **(A)** apoptosis- and **(B)** inflammation-related genes in comparison with doxorubicin. All data are expressed as the mean ± SEM; *n* = 3; *P < 0.05, **P < 0.01, ***P> < 0.001. One-way ANOVA was used to compare multiple independent groups using GraphPad Prism 7 software. *Dunnett’s and Tukey’s* multiple comparisons tests were used as post hoc tests.

Next, we investigated whether this combination may intensify the effects of apoptosis in Caco-2 cells through different key modulatory genes (*caspase-3*, *Cytochrome-C (Cyt-C)*-*Bax*,* Bcl-2* and *p53*) in addition to other indirectly related genes involved in inflammation (*NFkB* and *TNFα*) (Fig. 6). Compared with doxorubicin alone, the qRT‒PCR data demonstrated that the abietic acid‒doxorubicin combination therapy tended to enhance and have an additive effect on the expression of *caspase-3*, *Cyt-C*, *Bax* and *p53* with downregulation of *Bcl-2*. There was a significant difference *in the expression of the genes encoding caspase-3*,* Cyt-C*,* and Bax between the combination therapy group and the doxorubicin group (p < 0.001)*,* but there was no discernible difference in p53 expression between the two groups* (Fig. 6A). Moreover, Bax/Bcl-2 ratio showed 1.99 ± 0.4, 4.3 ± 0.8 and 17.6 ± 1.6 for abietic acid, doxorubicin and the combination respectively suggesting the efficacy of combination for induction of apoptosis. Additionally, Caco-2 cells treated with the combination therapy showed a similar trend for the inflammatory-related genes *NFkB* and *TNFα* (*P < 0.05* and *P* < 0.001, respectively) (Fig. 6B). These data collectively indicate that abietic acid and doxorubicin enhance the apoptotic impact in these cells through modulating proapoptotic factors directly and through other indirect factors.

### Overexpression of key apoptotic proteins (Cyt-c, Casp-3, p53 and Bax) with reduced BCL2 protein expressions were associated with augmented apoptosis in combination therapy

**Fig. 7:**
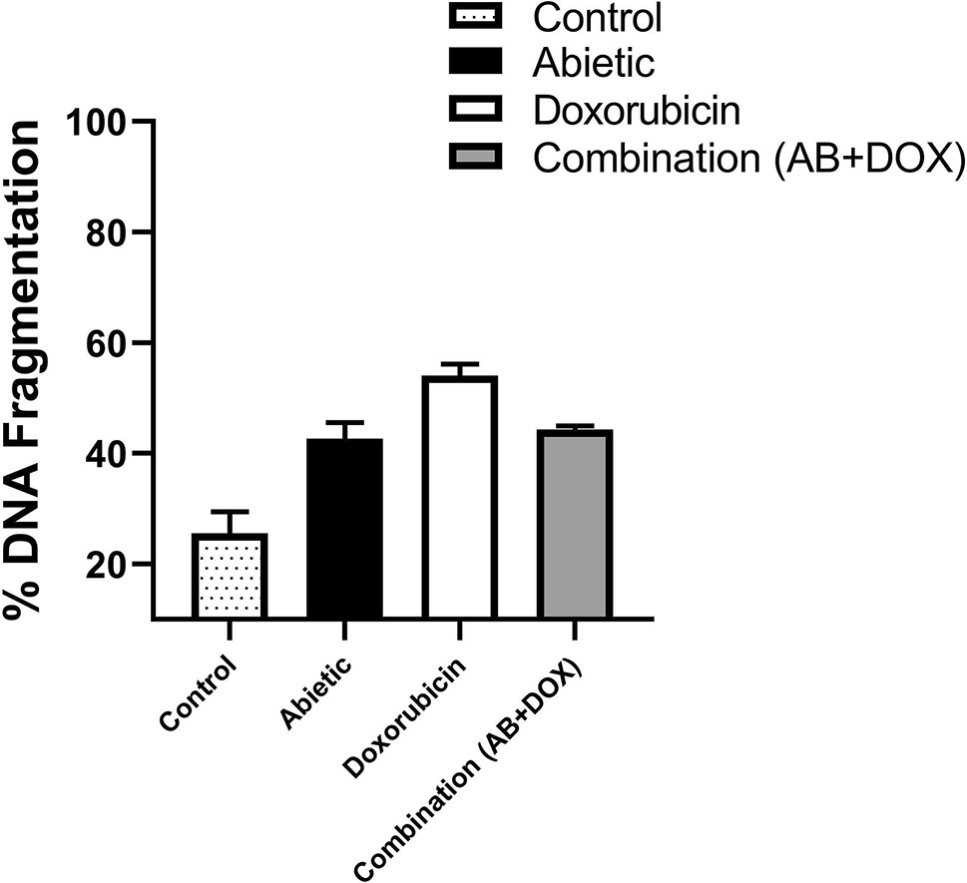
Quantification of DNA fragmentation in the control and experimental groups using a DPA assay. Bars represent DNA fragmentation (percentage of fragmented DNA to intact DNA) as the mean ± SEM; *n* = 3. There was no significant difference between the combination and doxorubicin groups according to one-way ANOVA. *Dunnett’s and Tukey’s* multiple comparisons tests were used as post hoc tests.

The suggested overexpression of the key apoptotic genes and downregulation of anti-apoptotic gene in combination therapy- treated group was explored on the protein level using ELISA assays. We have performed ELISA analysis to assess the protein levels of key markers, including Cyt-c, Casp-3, Bax, p53, and Bcl-2. Interestingly, the protein expression data were consistent with that of gene expression, further validating the findings and supporting the flow of our presented data (Fig. 7). Cyt-c is a key protein in the mitochondrial pathway of apoptosis, where it activates caspases and other proteins that drive the apoptotic cascade. A significant increase in Cyt-c levels in the combination therapy group was observed (10.78 ± 0.61) compared to negative control (2.9 ± 0.53, *P* < 0.0001), abietic acid (4.3 ± 0.66, *P* < 0.0001) and doxorubicin (6.6 ± 0.66, *P* < 0.0021) (Fig. 7). Casp-3 protein is an executioner caspase that plays a central role in the final stages of apoptosis. It is activated by both intrinsic and extrinsic apoptotic signals. Significant higher level of Casp-3 in the combination therapy group was observed (8.62 ± 0.48) compared to negative control (2.16 ± 0.13, *P* < 0.0001), abietic acid (4.96 ± 0.30, *P* < 0.0001) and doxorubicin (6.21 ± 0.28, *P* < 0.0010) (Fig. 7). Moreover, Bax is a pro-apoptotic protein that promotes mitochondrial outer membrane permeabilization, facilitating the release of pro-apoptotic factors like Cyt-c. Bax protein expression was significantly higher in the combination therapy (9.1 ± 0.34, *P* < 0.0001) compared to negative control (1.2 ± 0.1, *P* < 0.0001) and the individual drugs; abietic acid (2.2 ± 0.16, *P* < 0.0001) and doxorubicin (2.9 ± 0.1, *P* < 0.0001). Finally, Bcl-2 is an anti-apoptotic protein that inhibits cell death by preventing the release of pro-apoptotic factors like Cyt-c from mitochondria. A downregulation of Bcl-2 in the combination therapy group (1.9 ± 0.1, *P* < 0.0001), compared to control (7.2 ± 0.4, *P* < 0.0001), abietic acid (3.7 ± 0.2, *P* < 0.0035) and doxorubicin (3.6 ± 0.35, *P* < 0.0059) indicates that the anti-apoptotic signals are being diminished, which could allow for increased apoptosis. This is a desirable outcome in cancer therapy (Fig. 7). These data may suggest that the combination therapy may be more effective at tipping the balance towards apoptosis by inhibiting cell survival signals.

### Detection of cellular apoptosis by DNA fragmentation using combined therapy in Caco-2 cells

**Fig. 8:**
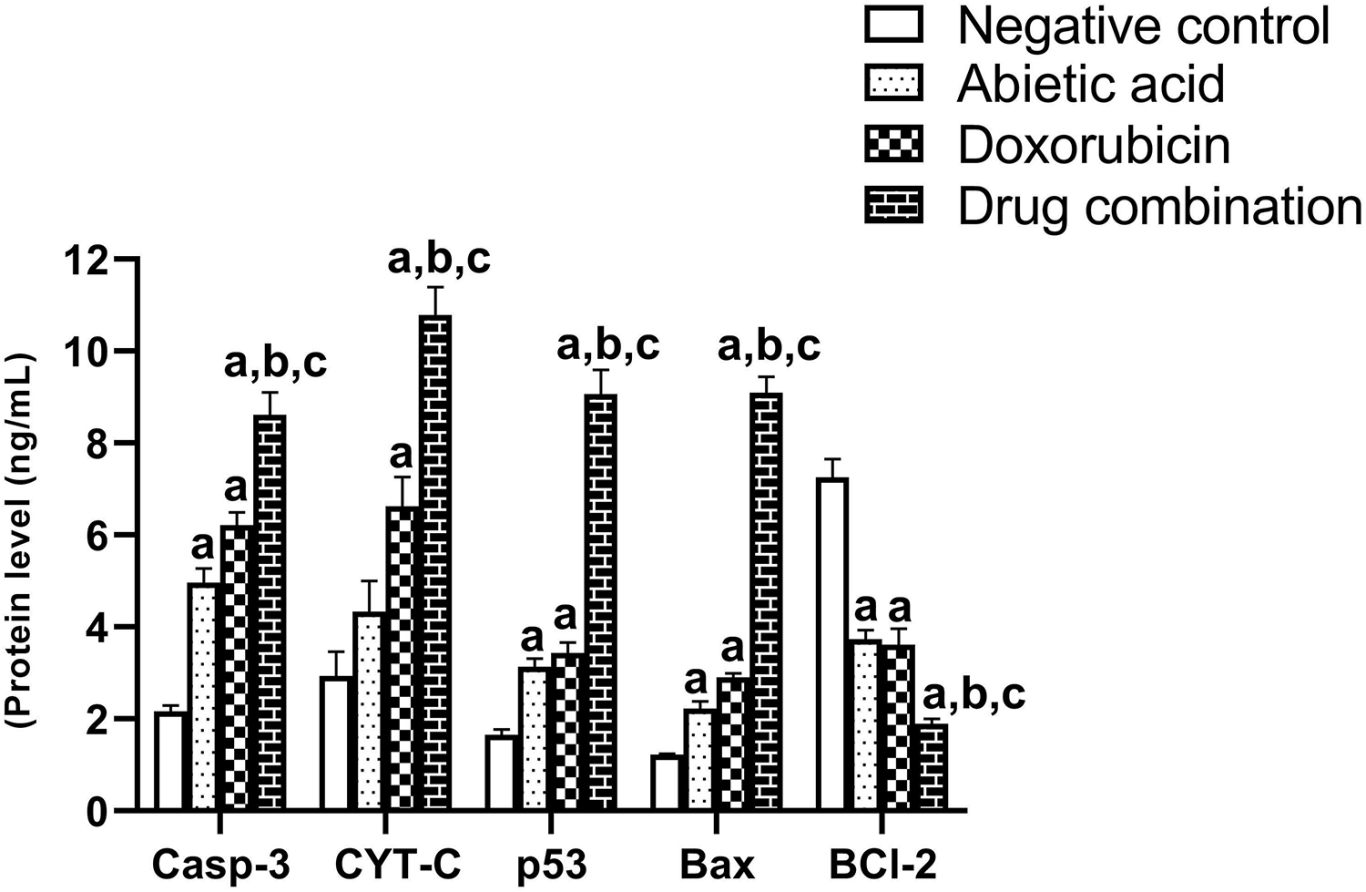
ELISA analysis of for the effects of abietic acid, doxorubicin and their combination in Caco-2 cells on the key apoptotic proteins (Casp-3, Cyt-C, p53, Bax and Bcl-2). The data is presented as protein quantity (ng/mL). All data are expressed as the mean ± SEM; *n* = 3; *P < 0.05, **P < 0.01, ***P < 0.001. *One-way ANOVA* was used to compare multiple independent groups using GraphPad Prism 7 software. *Dunnett’s and Tukey’s* multiple comparisons tests were used as post hoc tests. The significant difference compared to negative control group is denoted by ‘a’, significant difference compared to abietic acid group is denoted by ‘b’ while, significant difference compared to doxorubicin group is denoted by ‘c’.

Since DNA fragmentation is a sign of apoptotic cell death^103–105^, it was essential to assess the amount of DNA fragmentation in Caco-2 cells after single and combined therapy treatment at the IC_50_ for 24 h. Diphenylamine (DPA) was used to measure quantitative DNA fragmentation in Caco-2 cells via spectrophotometry. Abietic acid and doxorubicin groups induced 42.6% and 54.0% fragmented DNA, respectively, compared to the negative control group, which had less fragmented DNA (25.6%) (Fig. 8). Compared with doxorubicin, combination therapy resulted in considerable DNA fragmentation (44.3%), but the difference was not significant, suggesting that DNA fragmentation occurred at a relatively lower IC_50_ (Fig. 8).

### Combinatorial treatment with doxorubicin and abietic acid decreases the rate of wound closure of Caco-2 cell monolayers

**Fig. 9:**
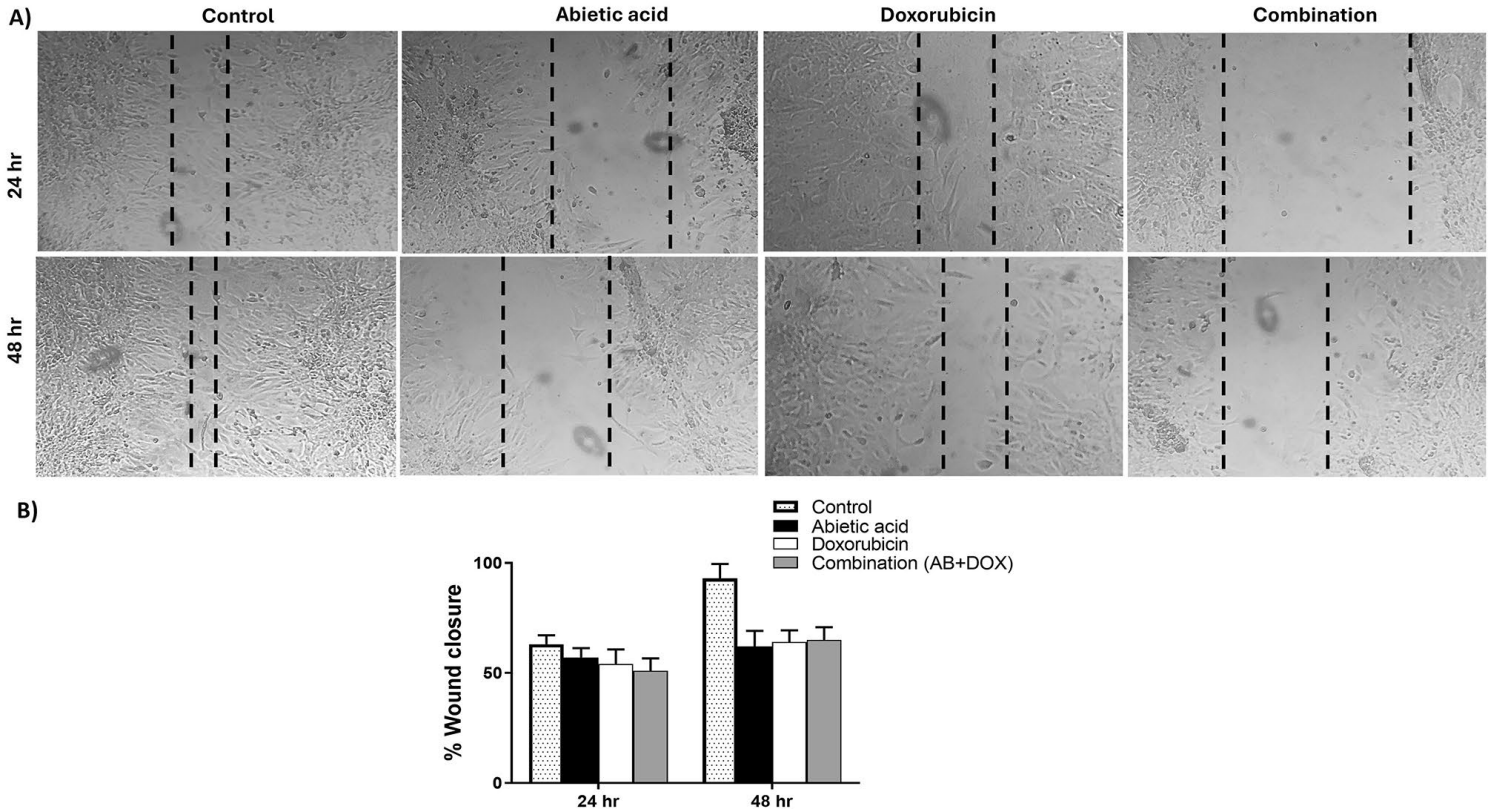
Wound healing assay over time showing (**A**) the impact of abietic acid, doxorubicin, and combination therapy on the capacity of Caco-2 cancer cells to migrate at 24 and 48 h. The percentage of wound closure was determined using ImageJ to measure (**B**) the migratory capacity, and the mean ± SEM of the independent experiments conducted in triplicate is presented as the percentage of the treated group compared to the untreated group as the % wound closure. Axio Observer inverted microscope at ×40 magnification. The mean ± SEM of the independent experiments conducted in triplicate are displayed as the quantitative results. There was no significant difference between the combination and doxorubicin groups according to one-way ANOVA. *Dunnett’s and Tukey’s* multiple comparisons tests were used as post hoc tests.

The impact of combination therapy on the migratory potential of highly metastatic Caco-2 cells was assessed using an in vitro scratch wound healing test since cell migration is a crucial component of metastasis and tumor invasion^106^. We used a scratch wound healing test to determine whether abietic acid-doxorubicin cotreatment can reduce the number of cells. Phase-contrast microscopy was used to assess the change of the percent of wound closure rate over temporal scale using ImageJ software. Figure 9A and Fig. 9B showed that negative untreated control showed wound closure rate of 63% after 24 h and 93% after 48 h suggesting the increase in metastatic effect of caco-2 cancer cells. Upon treatment with abietic acid, doxorubicin and the combination therapy for 24 h, the closure rates were 57%, 54% and 51% respectively which are lower than the closure rate of negative control at the same time point suggesting the reduction in the metastatic rate of caco-2. After 48 h of treatment, the closure rates were 62%, 64% and 65% for abietic acid, doxorubicin and combination therapy respectively which were much lower than the closure rate of negative control at the same time point. This may be explained as the combination therapy at lower doses was able to give similar effect as shown in abietic acid or doxorubicin on the wound closure rate. According to these findings, cotreating cells with abietic acid combined with doxorubicin reduces the extent to which the scratch wound closes in away similar to the treated cells with abietic acid or doxorubicin alone.

### HDACs inhibitory activity of combination therapy in Caco-2 cells

Next, we used Trichostatin (TSA) as the reference drug to examine the inhibitory efficacy and selectivity of both abietic acid, doxorubicin, and their combination for calculation of percentage inhibition of total Pan-HDAC isoforms activity. The results, which are displayed in Table 5, showed that at IC_50_ doses, TSA strongly suppressed the activity of total HDACs (91.17%) followed by abietic acid (84.7%) and finally doxorubicin (76.9%). Abietic acid and doxorubicin in combination had an inhibitory impact on HDAC activity (68.7%) that was less than the standard TSA and comparable to single compounds; however, this effect was obtained at lower IC_50_ doses of the compounds against different HDACs. Based on these findings, abietic acid seems to have an epigenetic role in combination with doxorubicin that specifically targets HDACs.

**Table 5:** Percentage Inhibition of HDACs of abietic acid, doxorubicin, and their combination in Caco-2 cell compared to TSA as positive control using their IC_50_ doses. The mean ± sd of the independent experiment conducted in triplicate was displayed as the quantitative results.

Tested compounds	% Total HDACs inhibitory activity
Trichostatin (TSA)	91.17 ± 6.4
Abietic	84.68 ± 3.8
Doxorubicin	76.89 ± 7.4
Abietic acid-doxorubicin combination	68.74 ± 6.9

## Discussion

One of the biggest obstacles in the chemotherapeutic treatment of cancer patients is inevitable acquired resistance and associated severe adverse effects^107^. Several molecular mechanisms are responsible for the progression of chemotherapeutic resistance, such as drug efflux, drug inactivation, reduced prodrug activation, drug target al.teration, growth factor alterations, extracellular matrix, microRNA and cytokine involvement, suppression of apoptosis, survival dysregulation, and hypoxia^108^. The majority of chemotherapy drugs are metabolized and eliminated by the kidney or liver, and hence they have a number of side effects that are indicative of their mode of action. These include tiredness, alopecia, myelosuppression, mucositis, nausea, vomiting, diarrhea, sterility, and infertility, as well as infusion responses^109–111^. Therefore, combination therapy is one of the useful strategy to enhance efficacy, reduce side effects and reduce cancer resistance. First, it lessens the likelihood of treatment resistance by combining medications that function through various pathways^2^. Second, this strategy targets several cancer growth stages and different molecular targets^112^. Also, combination therapy produces a synergistic effect which is greater than that of a single drug promoting efficacy of each drug, reducing toxic effects, and decreasing the amount of dose for each individual drug^113^. The combination with natural products and their derivatives is a good strategy than a single agent alone for anticancer drug development and have been employed to modulate several mechanisms and reducing associated side effects^114,115^. The current study showed the screening of 5-FU and doxorubicin as two examples of chemotherapeutic agents as well as two new molecules, abietic acid (from a natural source) and Raptinal (from a synthetic origin) in different cancer cell lines and normal fibroblast cell line (WI-38). Compared with doxorubicin and abietic acid, 5-FU had relatively lower IC_50_values and higher SI values in different cancer cell lines, except for PC-3, Caco-2, and HepG-2 cells. Although doxorubicin is a common cancer drug used to treat a range of human cancers^116^, its efficacy is reduced due to the overexpression of drug efflux transporters and proteins linked to multidrug resistance and cancer resistance proteins^116^. Moreover, various cancer cells resistant to doxorubicin frequently exhibit suppression of apoptosis^117^. This is accomplished by downregulating proapoptotic and upregulating antiapoptotic mediators^14^. Additionally, abietic acid showed significant anticancer efficacy as previously observed in our study on MCF-7 cancer cells with reduced cytotoxicity to normal cells and greater safety index compared to synthetic Raptinal^55^. Our previous research showed that abietic acid regulates many pathways, including the downregulation of oncogenic (NF-κB and C-myc) and proliferative (IGFR1, TGF-β and VEGF) mediators, to have adequate anticancer effects and activate apoptotic pathways^55^. Thus, it is critical to assess whether abietic acid amplifies the anticancer effects of doxorubicin on cancer cells and reduces its cytotoxicity as a novel combination of antiproliferative drugs. Caco-2 cell lines were selected for further investigations since colorectal cancer has a high rate of morbidity and death^118,119^, and doxorubicin is one of the most effective treatments for colorectal cancer with acquired resistance and toxicity. We assessed the IC_50_ of different chemotherapeutic agents (doxorubicin and 5-FU), as well as abietic acid and Raptinal, on Caco-2 cells using an isobologram protocol. Our data showed that the combination of the IC_50_ dose of abietic acid with doxorubicin was the only effective synergistic combination, reducing the IC_50_ to 30% of the original IC_50_, with minimal cytotoxicity to normal WI-38 cells. Additionally, CI analysis was carried out to determine how different combinations work in concert with colon cancer cells. The CI of abietic acid with doxorubicin (0.63) was the lowest, suggesting that abietic acid had a positive impact on the response of colon cancer cells to doxorubicin. Additionally, abietic acid produced a DRI value (3.286) in the range accepted for synergistic combinations that suppress cell growth at relatively low doses^120^. Our findings showed that Caco-2 cells treated with doxorubicin, abietic acid, or their combination exhibited morphological changes, including chromatin condensation, apoptotic body formation, and cell shrinkage—all of which are signs of apoptosis induction—with a much more pronounced effect on the combination than on cells treated with doxorubicin and abietic acid alone^121^. These findings coincided with numerous investigations have shown that when doxorubicin is combined with other naturally occurring substances such as hesperidin, naringin, ruthenium and berberine, its anticancer effects increase^106,122–124^. This may suggest that the anticancer effects of abietic acid-doxorubicin on Caco-2 cells are achieved by controlling a number of processes related to cell survival.

Through a series of actions that result in cell division and duplication, the cell cycle controls the machinery of cancer cells^125^. Cancer is caused by aberrant and uncontrolled cell proliferation, which is the outcome of cell cycle dysregulation^126^. Many anticancer agents act by causing cell cycle arrest at a particular checkpoint, including abietic acid, which was reported in our previous research to cause cell cycle arrest in MCF-7 cells at the G_2_/M and subG_0_-G_1_subpopulation phases^55^. Additionally, doxorubicin induces G_2_/M phase arrest in a wide range of cancer cells^127^. According to our data, abietic acid potentiates the ability of doxorubicin to cause the accumulation of cells in the G_2_/M phase of the cell cycle in the Caco-2 cell line and thus inhibits cell growth.

Additionally, apoptosis, or programmed cell death, is a crucial physiological process for the growth and homeostasis of tissues^128^, and one of the characteristics of cancer is the suppression of apoptosis^129^. It is linked to several alterations in the morphology of cells, including nuclear blebbing, DNA breakage, and chromatin condensation^130^, which were observed in this study. The amount of chromatin condensation and damage, which are characteristics of cell apoptosis, was measured by a DNA fragmentation assay. The combination protocol showed a pronounced DNA fragmentation effect at a relatively lower dose than abietic acid and doxorubicin treatments alone, which validated our findings. This observation matched other previous studies showing Flavokawain B and Doxorubicin Work Synergistically to induce DNA damage by ROS-Mediated Apoptosis and Autophagy Pathways^131^and Artesunate induce DNA damage with doxorubicin in Doxorubicin-Resistant T Leukemia Cells by induction of ROS-mediated apoptosis^132^. In the present study, flow cytometry was used to determine how combination treatment affects both the cell membrane and nuclear damage, and Annexin-V/PI was used to determine the proportions of living and dead cells^128^. The current study revealed that, whereas the abietic acid, doxorubicin, and combination treatments decreased the number of viable cells and increased the number of apoptotic cells, the number of viable cells was greatest for the untreated cells. There was a tiny increase in cell viability after abietic acid treatment that could be explained as abietic acid is a natural diterpenoid, and it has been reported in some studies to exhibit bioactive properties that may influence cell viability, including cytoprotective anti-inflammatory and antioxidant effects under the specific experimental conditions used in this study^133–136^. Hence, this was one of the major aims of the study to use natural product compound that enhance activity of chemotherapeutic drugs without exerting additional cytotoxicity to cells. A greater proportion of cells in the combination treatment group than in the abietic acid and doxorubicin treatment groups exhibited both late apoptosis and necrosis at relatively lower doses. This observation matched with previous studies showed that doxorubicin combination therapy had enhanced effect at relatively low doses causing more than 90% late apoptosis/necrosis^137,138^. The possible explanation is the combined mechanisms of DNA fragmentation, ROS mediated apoptosis, cell membrane blebbing and reduced cell viability leads to enhanced activity shown in abietic acid-doxorubicin combination with minimal toxicity on normal cells as observed in our study. Interestingly, while doxorubicin alone is a potent cytotoxic agent that significantly reduces cell viability, the addition of abietic acid may partially counteract this effect by mitigating doxorubicin-induced stress or apoptosis in a subset of cells. This could result in a modest but non-significant increase in viable cells compared to doxorubicin alone. On the other hand, the combination therapy was able to induce late necrosis (UR). This suggests that the combination of abietic acid and doxorubicin may alter the mode of cell death, shifting it from apoptosis (which is more characteristic of doxorubicin alone) to necrosis. This shift could be due to abietic acid’s influence on cellular stress pathways, mitochondrial function, or membrane integrity, leading to a different cell death phenotype. On contrast, Abietic acid, demonstrated less apoptotic effect for the Caco-2 cells despite its previously documented antiproliferative activity and early apoptosis on MCF-7 ^55^. This could be attributed to the effect specific to cancer cell type, and antiproliferation effect can result from cell cycle arrest and cell senescence with apoptosis-independent cell death^139^, which can be complemented by other target-specific mechanisms as previously shown in literature^138,140^. The hypothesis was also confirmed on HCT-116 colorectal cell line that is known for its chemotherapeutic resistance^141,142^. The combination therapy was able to induce both synergistic apoptotic effect as well as cell cycle arrest matched with previously observed literature for combination therapy Curcumol with 5-FU^143^and Metformin, Sodium Oxamate with Doxorubicin^144^. Thus, these findings provide additional evidence that an apoptotic mechanism mediates the cytotoxic effects of abietic acid-doxorubicin combination.

Numerous stress signals, including DNA damage and an aberrant cell cycle, trigger the intrinsic apoptotic pathway. It is controlled by genes and proteins that are both pro- and antiapoptotic, and it triggers the cascade of caspases that kill these cells^145^. To elucidate the mechanism of abietic acid, doxorubicin, and their combination, the route of action of the essential key regulatory signals of apoptosis (*caspase-3*, *Bax*, *Cyt-c*, *Bcl-2* and *p53*) was investigated. The key apoptotic markers caspase-3, Bax (extra-mitochondrial membrane marker)^146,147^, p53 (extra mitochondrial membrane marker)^148,149^, Cyt-c (intra-mitochondrial membrane marker)^150,151^are used for evaluation. Caspase-3 is known as central executioner marker for apoptosis and responsible for the initiation of both intrinsic and extrinsic pathways^152,153^. Therefore, it was interesting for us to assess the modulation of these markers on gene level. The expression of *caspase-3*, *Bax*, *Cyt-c* and *p53* proapoptotic genes increased while *Bcl-2*gene was downregulated. The Bax/Bcl-2 ratio was 17.6-fold with combination therapy and greater than control, abietic acid and doxorubicin matched with previously observed anticancer agents^77,78^. This may indicate combination therapy enhances cell’s capacity to react to an apoptotic signal confirmed by the intracellular Bax/Bcl-2 ratio that acts as prognostic indicator for tumor aggressiveness, progression and assesses a cell’s propensity for apoptosis^74–76,154,155^. This genetic modulation effect was reflected in the increase in the expression of their corresponding proteins (Cyt-C, Casp-3, p53, Bax) with decrease in expression of Bcl-2 protein with significant difference in expression level between doxorubicin or its combination. This may be explained as the combination therapy at lower doses was able to give similar effect shown in doxorubicin on the expression level of proteins to improve the response of Caco-2 cells to doxorubicin by enhancing the ability of cells to undergo apoptosis. It worth to mention that although caspase-3 is a cytosolic protein that mediates apoptosis in caco-2 cells, it would be better to measure the proteolytic change in caspase-3 and other key apoptotic mediators in the future as a reflection for signal transduction that ultimately leads to apoptosis^156^and as a prognostic biomarker for tumor stages and cell differentiation status following combination treatment^157^. Also, it worth to measure other key proteolytic intermediate protein mediators in the future as a reflection for signal transduction that ultimately leads to apoptosis^156^and as a prognostic biomarker for tumor stages and cell differentiation status following combination treatment^157^.

It worth to mention that doxorubicin did not show a significant effect on both *TNF-α* and the *NF-κB*genes. The response to doxorubicin can vary significantly depending on the cell line-specific response^158^. Caco-2 cells, which were employed in this study, may exhibit inherent resistance or alter sensitivity to doxorubicin due to differences in drug uptake, efflux mechanisms and intracellular signaling pathways^159–161^. For example, some studies have reported that Caco-2 cells express high levels of drug efflux transporters such as P-glycoprotein, which could reduce intracellular doxorubicin concentrations and attenuate its apoptotic effects^162–164^. Therefore, the NF-κB and TNFα gene expression level with doxorubicin treatment only were reduced but overexpressed after combinatorial therapy suggesting the synergistic effect. Also, the concentration of doxorubicin and the duration of treatment for 24 h used in our study were optimized to evaluate combination effects with abietic acid. These conditions may not have been sufficient to fully induce the expected NF-κB and TNFα gene expression level response. It is possible that higher concentrations or longer exposure times would be required to observe significant apoptosis in this model. Moreover, while ROS, NF-κB and TNFα induction are primary mechanisms of doxorubicin-induced cell death, our data suggest a shift toward other mechanisms of action as induction of apoptosis through intrinsic mitochondrial pathway supported by our data for overexpression of key apoptotic genes (Cyt-C, p53, Casp-3, Bax and Bcl-2) matched with other studies^165–167^. Also, the necrosis observed in the combination treatment group may indicate that the presence of abietic acid alters the cellular response to doxorubicin, potentially through modulation of NF-κB and TNFα levels, mitochondrial function, or other stress pathways. These findings suggest that the effect of doxorubicin on NF-κB and activation can vary depending on different factors. On the other hand, the abietic acid-doxorubicin combination was shown to enhance the expression of TNF-α and the NF-κB signalling pathway in contrast to doxorubicin. TNF-α functions as a proinflammatory cytokine that is involved in inflammation and cancer, including cellular differentiation, proliferation, and apoptosis^168,169^. Its tumorigenic activity is mediated by activating the proinflammatory transcription factor NF-κB, which induces the expression of genes related to the progression and development of cancer, including (i) antiapoptotic genes such as Bcl-2, Bcl-xL, Survivin, and c-IAP1/2; (ii) invasive genes (MMP-9, ICAM-1, and VCAM-1); (iii) growth factors (Cyclin D1, c-MYC, interleukin 1, and interleukin 6; and (iv) angiogenic factors (VEGF)^170,171^. However, TNF-α was identified in other studies as an anticancer factor because of its exceptional capacity to elicit apoptosis in human malignant cells while sparing normal cells^169,172^. The possible mechanism includes binding to cellular TNF-α receptor 1 (TNFR1), which causes cancer cells to undergo apoptosis by activating reactive oxygen species (ROS) and releasing Cyt-C from mitochondria, or by attracting TNFR-associated death domain (TRADD) and Fas-associated protein with death domain (FADD)^173–176^. Considering this mechanism, combination therapy involving TNF-α and NF-κB activation as well as elevated Cyt-C can sensitize Caco-2 cells to boost TNF-α-mediated apoptosis and inflammation to cure cancer.

Moreover, previous research has shown that many cancer cell types have an elevated HDAC activity, and inhibiting this activity would be a suitable therapeutic target for the treatment of cancer^177,178^. It was reported that standard HDAC inhibitors such as suberoylanilide hydroxamic acid (SAHA) stimulates both intrinsic and extrinsic apoptosis pathways. Additionally, SAHA induces G_2_M phase arrest after 24 h and increases the number of sub-G1 cells, annexin V-FITC positive cells, and mitochondrial membrane potential (MMP) loss, reduction in Bcl-2 level, and increasing cleavage of PARP, Bax, and caspase-3 activity^179^. Our data showed abietic acid was able to inhibit HDAC activity in away similar to Trichostatin standard HDAC inhibitor and much more than doxorubicin and combination therapy was able to enhance HDAC inhibitory activity to 68.74% at lower dose matched with all previously mentioned inhibitory activity on growth of Caco-2 cells.

Colorectal cancer patients have a high mortality rate because of tumor development and metastasis to distant regions^180^. Compared to abietic acid or doxorubicin monotherapy, wound healing experiments showed that the invasion and migration rate of combination-treated Caco-2 cells were dramatically reduced at relatively lower doses in a time-dependent manner. Previous study showed naringin combined with doxorubicin had a synergetic inhibitory influence on MCF-7 cells migration^122^. Also, Oxymatrine showed significant inhibition of cell migration with co-treatment with doxorubicin in colorectal cancer cells^181^. Moreover, benzophenanthridine alkaloid sanguinarine, chelerythrine and chelidonine inhibited cell migration at concentrations below apparent cytotoxicity in co-treatment with doxorubicin^182^. All these studies were consistent with the observed effect of our study for the combination therapy on reducing cell proliferation DNA damage, reducing cellular repair and enhancing cell death, which are all reflected in the rate of wound healing closure. The current study suggests a new combination chemotherapy strategy for the management of Caco-2 cancer cells as example for colorectal cancer that deserve trial on all other cancer types. It was demonstrated that abietic acid inhibited the invasiveness of Caco-2 cells and increased the anticancer effect of doxorubicin by inducing apoptosis. Nevertheless, further research in future is needed to determine the anticancer efficacy of abietic acid-doxorubicin combination on both in-vivo and clinical level as supplement to effectively manage colorectal cancer patients with prescribed chemotherapeutic drugs at lower doses. Also, studying the modulation in the proteolytic activity of several markers may give indication for signal transduction activity mediated by abietic acid for several pathways. Also, it worth to test the effect of this combinatorial regimen on the protein level of several ket regulatory proteins. Finally, it may be interesting to test the epigenetic effect of the combination therapy on some key modulatory markers for epigenetics such as Histone deacetylases (HDACs) and DNA methyltransferases (DNMT’s).

## Conclusion

The combination of abietic acid with doxorubicin notably reduced the viability of Caco-2 colorectal cancer cells in a synergistic manner. Moreover, the combined therapy caused apoptotic cell death because it changed the morphology of the cells, induced DNA fragmentation, reduced wound healing closure, and stimulated the expression of apoptotic markers (caspase-3, Bax, Cyt-C and p53) with reduction of Bcl-2 at both gene and protein levels as well as inflammatory inducers (*TNF-α and NF-κB*). We suggest that using abietic acid with doxorubicin has promising antiproliferative and apoptotic properties and can be used for manging colorectal cancer cells. Nonetheless, further research is needed, particularly regarding the molecular process of apoptotic cell death by investigating western blotting analysis for comparative analysis with ELISA data for some key apoptotic markers such as caspase-3 as well as some of key regulatory proteins and epigenetic markers such as HDACs and DNMT’s are of valuable roles to understand the combination effect of the drugs. Finally, in-vivo and clinical studies will be of substantial interest to open the window for new therapeutic approaches for management of colorectal and other cancer cell types.

## Electronic supplementary material

Below is the link to the electronic supplementary material.

Supplementary Material 1

Supplementary Material 2

Supplementary Material 3

Supplementary Material 4

Supplementary Material 5

Supplementary Material 6

Supplementary Material 7

Supplementary Material 8

Supplementary Material 9

Supplementary Material 10

Supplementary Material 11

## Data Availability

All data generated or analysed during this study are included in this published article [and its supplementary information files]. Any further datasets generated during and/or analysed during the current study are available from the corresponding author on reasonable request.

## Abbreviations

5-FU: 5-Fluorouracil
CRC: Colorectal Cancer
NPs: Natural products
CI: Combination index
DRI: Dose reduction index
AV: Annexin V
PI: Propidium Iodide
HDACs: Histone deacetylases
TNFR1: TNF-α receptor 1
TRADD: TNFR-associated death domain
FADD: Fas-associated protein with death domain

## References

[1] Nagai, H. & Kim, Y. H. Cancer prevention from the perspective of global cancer burden patterns. *J. Thorac. Dis.* **9**, 448 (2017). 28449441 10.21037/jtd.2017.02.75 PMC5394024

[2] Mokhtari, R. B. et al. Combination therapy in combating cancer. *Oncotarget* **8**, 38022 (2017). 28410237 10.18632/oncotarget.16723 PMC5514969

[3] Debela, D. T. et al. New approaches and procedures for cancer treatment: current perspectives. *SAGE Open. Med.* **9**, 20503121211034366 (2021). 34408877 10.1177/20503121211034366 PMC8366192

[4] Chehelgerdi, M. et al. Progressing nanotechnology to improve targeted cancer treatment: overcoming hurdles in its clinical implementation. *Mol. Cancer*. **22**, 169 (2023). 37814270 10.1186/s12943-023-01865-0 PMC10561438

[5] Barrios, C. H. Global challenges in breast cancer detection and treatment. *Breast* **62**, S3–S6 (2022). 35219542 10.1016/j.breast.2022.02.003 PMC9097801

[6] Pucci, C., Martinelli, C. & Ciofani, G. Innovative approaches for cancer treatment: Current perspectives and new challenges. *ecancermedicalscience* 13 (2019). 10.3332/ecancer.2019.961 PMC6753017 31537986

[7] Fox, P. et al. The assessment and management of chemotherapy-related toxicities in patients with breast cancer, colorectal cancer, and Hodgkin’s and non-Hodgkin’s lymphomas: A scoping review. *Eur. J. Oncol. Nurs.* **26**, 63–82 (2017). 28069154 10.1016/j.ejon.2016.12.008

[8] Damodar, G., Smitha, T., Gopinath, S., Vijayakumar, S. & Rao, Y. An evaluation of hepatotoxicity in breast cancer patients receiving injection doxorubicin. *Annals Med. Health Sci. Res.* **4**, 74–79 (2014). 10.4103/2141-9248.126619 PMC3952301 24669335

[9] Chakraborty, S. & Rahman, T. The difficulties in cancer treatment. *Ecancermedicalscience* **6** (2012). 10.3332/ecancer.2012.ed16 PMC4024849 24883085

[10] Moiseeva, A. Anthracycline derivatives and their anticancer activity. *Apoptosis* **25**, 26 (2019).

[11] Peter, S., Alven, S., Maseko, R. B. & Aderibigbe, B. A. Doxorubicin-based hybrid compounds as potential anticancer agents: a review. *Molecules* **27**, 4478 (2022). 35889350 10.3390/molecules27144478 PMC9318127

[12] Mattioli, R. et al. Doxorubicin and other anthracyclines in cancers: activity, chemoresistance and its overcoming. *Mol. Aspects Med.* **93**, 101205 (2023). 37515939 10.1016/j.mam.2023.101205

[13] Ramos, A., Sadeghi, S. & Tabatabaeian, H. Battling chemoresistance in cancer: root causes and strategies to uproot them. *Int. J. Mol. Sci.* **22**, 9451 (2021). 34502361 10.3390/ijms22179451 PMC8430957

[14] Cox, J. & Weinman, S. Mechanisms of doxorubicin resistance in hepatocellular carcinoma. *Hepatic Oncol.* **3**, 57–59 (2016). 10.2217/hep.15.41 PMC4792121 26998221

[15] Li, Q. & Wang, L. Autophagy, Paradoxical functions and perspective in cancer treatment. *Oncol. Cancer Case Rep.* **1**, 1–4 (2015).

[16] Zhang, N., Yin, Y., Xu, S. J. & Chen, W. S. 5-Fluorouracil: mechanisms of resistance and reversal strategies. *Molecules* **13**, 1551–1569 (2008). 18794772 10.3390/molecules13081551 PMC6244944

[17] Jubeen, F. et al. Anticancer potential of novel 5-fluorouracil co-crystals against MCF7 breast and SW480 colon cancer cell lines along with Docking studies. *Arab. J. Chem.* **15**, 104299 (2022).

[18] Alzahrani, S. M., Doghaither, A., Al–Ghafari, H. A., Pushparaj, P. & A. B. & N. 5–Fluorouracil and capecitabine therapies for the treatment of colorectal cancer. *Oncol. Rep.* **50**, 1–16 (2023). 10.3892/or.2023.8612 37594133

[19] Entezar-Almahdi, E., Mohammadi-Samani, S., Tayebi, L. & Farjadian, F. Recent advances in designing 5-fluorouracil delivery systems: a stepping stone in the safe treatment of colorectal cancer. *Int. J. Nanomed.*, 5445–5458 (2020). 10.2147/IJN.S257700 PMC7398750 32801699

[20] Palchaudhuri, R. et al. A small molecule that induces intrinsic pathway apoptosis with unparalleled speed. *Cell. Rep.* **13**, 2027–2036 (2015). 26655912 10.1016/j.celrep.2015.10.042 PMC4683402

[21] Haffez, H., Taha, H., Farrag, N. S., Amin, A. M. & Hassan, Z. A. Biological screening and radiolabeling of raptinal as a potential anticancer novel drug in hepatocellular carcinoma model. *Eur. J. Pharm. Sci.* **158**, 105653 (2021). 33253882 10.1016/j.ejps.2020.105653

[22] Santavanond, J. P. et al. The small molecule raptinal can simultaneously induce apoptosis and inhibit PANX1 activity. *Cell. Death Dis.* **15**10.1038/s41419-024-06513-z (2024). 10.1038/s41419-024-06513-z PMC10858176 38336804

[23] Wu, J., Li, Y., He, Q. & Yang, X. Exploration of the use of natural compounds in combination with chemotherapy drugs for tumor treatment. *Molecules* **28**, 1022 (2023). 36770689 10.3390/molecules28031022 PMC9920618

[24] Sun, X. et al. NPCDR: natural product-based drug combination and its disease-specific molecular regulation. *Nucleic Acids Res.* **50**, D1324–D1333 (2022). 34664659 10.1093/nar/gkab913 PMC8728151

[25] Zhang, Y. et al. The combinatory effects of natural products and chemotherapy drugs and their mechanisms in breast cancer treatment. *Phytochem Rev.* **19**, 1179–1197 (2020).

[26] Asma, S. T. et al. Natural products/bioactive compounds as a source of anticancer drugs. *Cancers (Basel)*. **14**, 6203 (2022). 36551687 10.3390/cancers14246203 PMC9777303

[27] Cragg, G. M. & Pezzuto, J. M. Natural products as a vital source for the discovery of cancer chemotherapeutic and chemopreventive agents. *Med. Princ Pract.* **25**, 41–59 (2016). 26679767 10.1159/000443404 PMC5588531

[28] Dasari, S., Njiki, S., Mbemi, A., Yedjou, C. G. & Tchounwou, P. B. Pharmacological effects of cisplatin combination with natural products in cancer chemotherapy. *Int. J. Mol. Sci.* **23**, 1532 (2022). 35163459 10.3390/ijms23031532 PMC8835907

[29] Aghababaei, F. & Hadidi, M. Recent advances in potential health benefits of Quercetin. *Pharmaceuticals* **16**, 1020 (2023). 37513932 10.3390/ph16071020 PMC10384403

[30] Nisar, S. et al. Natural products as chemo-radiation therapy sensitizers in cancers. *Biomed. Pharmacother*. **154**, 113610 (2022). 36030591 10.1016/j.biopha.2022.113610

[31] Mansouri, K. et al. Clinical effects of Curcumin in enhancing cancer therapy: A systematic review. *BMC Cancer*. **20**, 1–11 (2020). 10.1186/s12885-020-07256-8 PMC7446227 32838749

[32] Salehi, B. et al. Therapeutic potential of Quercetin: new insights and perspectives for human health. *Acs Omega*. **5**, 11849–11872 (2020). 32478277 10.1021/acsomega.0c01818 PMC7254783

[33] Xing, K. & Jiang, W. Abietic acid ameliorates neuroinflammation and blood-brain barrier disruption in traumatic brain injury by inhibiting MAPK pathway. *Trop. J. Pharm. Res.* **21**, 1893–1897 (2022).

[34] Yang, N. & Tian, L. Preventive effect of abietic acid against skin Cancer of mice. *Nat. Prod. Commun.* **12**, 1934578X1701200904 (2017).

[35] Liu, X., Chen, W., Liu, Q. & Dai, J. Abietic acid suppresses non-small-cell lung cancer cell growth via blocking IKKβ/NF-κB signaling. *Onco Targets Ther.*, 4825–4837 (2019). 10.2147/OTT.S199161 PMC6590626 31354305

[36] Ali, G., Ara, T. & Design Synthesis, and in vitro anticancer activity of Triazolyl analogs of abietic acid. *Russ J. Org. Chem.* **59**, 142–149 (2023).

[37] Elfadadny, A. et al. Natural bioactive compounds-doxorubicin combinations targeting topoisomerase II-alpha: anticancer efficacy and safety. *Toxicol. Appl. Pharmacol.*, 116405 (2023). 10.1016/j.taap.2023.116405 36716865

[38] Guerriero, E. et al. Combining doxorubicin with a phenolic extract from flaxseed oil: evaluation of the effect on two breast cancer cell lines. *Int. J. Oncol.* **50**, 468–476 (2017). 28101573 10.3892/ijo.2017.3835

[39] Lee, M. G., Lee, K. S. & Nam, K. S. Combined doxorubicin and Arctigenin treatment induce cell cycle arrest-associated cell death by promoting doxorubicin uptake in doxorubicin‐resistant breast cancer cells. *IUBMB Life*. **75**, 765–777 (2023). 37492896 10.1002/iub.2772

[40] Kamran, S., Sinniah, A., Chik, Z. & Alshawsh, M. A. Diosmetin exerts synergistic effects in combination with 5-fluorouracil in colorectal cancer cells. *Biomedicines* **10**, 531 (2022). 35327333 10.3390/biomedicines10030531 PMC8945009

[41] Nobari, S., Najafi, R., Mahdavinezhad, A. & Amini, R. The combination of zerumbone with 5-fluorouracil for sensitizing colorectal cancer-associated fibroblasts to treatment. *Evid. Based Complement. Alternat. Med.* (2022). (2022). 10.1155/2022/9369328 PMC9017496 35449812

[42] Ndreshkjana, B. et al. Combination of 5-fluorouracil and thymoquinone targets stem cell gene signature in colorectal cancer cells. *Cell. Death Dis.* **10**, 379 (2019). 31097715 10.1038/s41419-019-1611-4 PMC6522523

[43] Mafi, A. et al. Melatonin and 5-fluorouracil combination chemotherapy: opportunities and efficacy in cancer therapy. *Cell. Communication Signal.* **21**, 33 (2023). 10.1186/s12964-023-01047-x PMC9912526 36759799

[44] Qayum, A. et al. Synergistic combination of PMBA and 5-fluorouracil (5-FU) in targeting mutant KRAS in 2D and 3D colorectal cancer cells. *Heliyon* **8** (2022). 10.1016/j.heliyon.2022.e09103 PMC9014391 35445157

[45] Jenke, R., Reßing, N., Hansen, F. K., Aigner, A. & Büch, T. Anticancer therapy with HDAC inhibitors: mechanism-based combination strategies and future perspectives. *Cancers (Basel)*. **13**, 634 (2021). 33562653 10.3390/cancers13040634 PMC7915831

[46] Hull, E. E., Montgomery, M. R. & Leyva, K. J. HDAC inhibitors as epigenetic regulators of the immune system: impacts on cancer therapy and inflammatory diseases. *BioMed research international* (2016). (2016). 10.1155/2016/8797206 PMC4983322 27556043

[47] Zhang, G. & Gan, Y. H. Synergistic antitumor effects of the combined treatment with an HDAC6 inhibitor and a COX-2 inhibitor through activation of PTEN. *Oncol. Rep.* **38**, 2657–2666 (2017). 29048666 10.3892/or.2017.5981 PMC5780018

[48] Palmer, A. C. & Sorger, P. K. Combination cancer therapy can confer benefit via patient-to-patient variability without drug additivity or synergy. *Cell* **171**, 1678–1691 (2017). e1613. 29245013 10.1016/j.cell.2017.11.009 PMC5741091

[49] Jardim, D. L., De Melo Gagliato, D., Nikanjam, M., Barkauskas, D. A. & Kurzrock, R. Efficacy and safety of anticancer drug combinations: a meta-analysis of randomized trials with a focus on immunotherapeutics and gene-targeted compounds. *Oncoimmunology* **9**, 1710052 (2020). 32002305 10.1080/2162402X.2019.1710052 PMC6959453

[50] Chen, K., Huang, Y. & Chen, J. -l. Understanding and targeting cancer stem cells: therapeutic implications and challenges. *Acta Pharmacol. Sin*. **34**, 732–740 (2013). 23685952 10.1038/aps.2013.27 PMC3674516

[51] Ayoub, N. (2021).

[52] Delou, J. M., Souza, A. S., Souza, L. C. & Borges, H. L. Highlights in resistance mechanism pathways for combination therapy. *Cells* **8**, 1013 (2019). 31480389 10.3390/cells8091013 PMC6770082

[53] Abdelaal, M. R., Soror, S. H., Elnagar, M. R. & Haffez, H. Revealing the potential application of EC-synthetic retinoid analogues in anticancer therapy. *Molecules* **26**, 506 (2021). 33477997 10.3390/molecules26020506 PMC7835894

[54] Bullying et al. Augmented therapeutic potential of EC-Synthetic retinoids in Caco-2 Cancer cells using an in vitro approach. *Int. J. Mol. Sci.* **23**, 9442 (2022). 36012706 10.3390/ijms23169442 PMC9409216

[55] Haffez, H., Osman, S., Ebrahim, H. Y. & Hassan, Z. A. Growth Inhibition and apoptotic effect of pine extract and abietic acid on MCF-7 breast cancer cells via alteration of multiple gene expressions using in vitro approach. *Molecules* **27**, 293 (2022). 35011526 10.3390/molecules27010293 PMC8746537

[56] Khedr, M. A. et al. Novel Thienopyrimidine analogues as potential metabotropic glutamate receptors inhibitors and anticancer activity: synthesis, In-vitro, In-silico, and SAR approaches. *Bioorg. Chem.* **109**, 104729 (2021). 33676314 10.1016/j.bioorg.2021.104729

[57] Sarhan, M. O., Haffez, H., Elsayed, N. A., El-Haggar, R. S. & Zaghary, W. A. New phenothiazine conjugates as apoptosis inducing agents: design, synthesis, In-vitro anti-cancer screening and 131I-radiolabeling for in-vivo evaluation. *Bioorg. Chem.* **141**, 106924 (2023). 37871390 10.1016/j.bioorg.2023.106924

[58] Haffez, H. et al. Biological evaluation and molecular Docking studies of novel thiopyrimidine analogue as apoptotic agent with potential anticancer activity. *Bioorg. Chem.*, 104249 (2020). 10.1016/j.bioorg.2020.104249 32911199

[59] Ebrahim, H. Y., Osman, S. A., Haffez, H. R. & Hassan, Z. A. In-vitro screening of some plant extracts for their potential anticancer activity. *Afr. J. Tradit. Complement. Altern. Med.* **17**, 1–8 (2020).

[60] Volpe, D. A., Hamed, S. S. & Zhang, L. K. Use of different parameters and equations for calculation of IC 50 values in efflux assays: potential sources of variability in IC 50 determination. *AAPS J.* **16**, 172–180 (2014). 24338112 10.1208/s12248-013-9554-7 PMC3889528

[61] Lyles, R. H., Poindexter, C., Evans, A., Brown, M. & Cooper, C. R. Nonlinear model-based estimates of IC50 for studies involving continuous therapeutic dose–response data. *Contemp. Clin. Trials*. **29**, 878–886 (2008). 18582601 10.1016/j.cct.2008.05.009 PMC2586183

[62] Peña-Morán, O. A., Villarreal, M. L., Álvarez-Berber, L., Meneses-Acosta, A. & Rodríguez-López, V. Cytotoxicity, post-treatment recovery, and selectivity analysis of naturally occurring Podophyllotoxins from Bursera fagaroides Var. fagaroides on breast cancer cell lines. *Molecules* **21**, 1013 (2016). 27527135 10.3390/molecules21081013 PMC6274026

[63] Braga, C. B. et al. Enhancing the anticancer activity and selectivity of goniothalamin using pH-Sensitive acetalated dextran (Ac-Dex) nanoparticles: A promising platform for delivery of natural compounds. *ACS Biomaterials Sci. Eng.* **6**, 2929–2942 (2020). 10.1021/acsbiomaterials.0c00057 33463303

[64] Ye, L., Tao, K., Yu, Y. & Wang, G. Reduction of G0 phase cells of colon cancer caco-2 cells May enhance 5-fluorouracil efficacy. *J. Biomedical Res.* **24**, 64–68 (2010). 10.1016/S1674-8301(10)60010-3 PMC3596537 23554613

[65] Handali, S. et al. A novel 5-Fluorouracil targeted delivery to colon cancer using folic acid conjugated liposomes. *Biomed. Pharmacother.* **108**, 1259–1273 (2018). 30372827 10.1016/j.biopha.2018.09.128

[66] Chou, T. C. & Talalay, P. Generalized equations for the analysis of inhibitions of Michaelis-Menten and higher-order kinetic systems with two or more mutually exclusive and nonexclusive inhibitors. *Eur. J. Biochem.* **115**, 207–216. 10.1111/j.1432-1033.1981.tb06218.x (1981). 7227366 10.1111/j.1432-1033.1981.tb06218.x

[67] Chou, T. C. & Talalay, P. Quantitative analysis of dose-effect relationships: the combined effects of multiple drugs or enzyme inhibitors. *Adv. Enzyme Regul.* **22**, 27–55. 10.1016/0065-2571(84)90007-4 (1984). 6382953 10.1016/0065-2571(84)90007-4

[68] Huang, L., Jiang, Y. & Chen, Y. Predicting drug combination index and simulating the network-regulation dynamics by mathematical modeling of drug-targeted EGFR-ERK signaling pathway. *Sci. Rep.* **7**, 40752 (2017). 28102344 10.1038/srep40752 PMC5244366

[69] Wali, V. B. & Sylvester, P. W. Synergistic antiproliferative effects of γ-tocotrienol and Statin treatment on mammary tumor cells. *Lipids* **42**, 1113–1123 (2007). 17701065 10.1007/s11745-007-3102-0

[70] Erdem, S. S., Obeidin, V. A., Yigitbasi, T., Tumer, S. S. & Yigit, P. Verteporfin mediated sequence dependent combination therapy against ovarian cancer cell line. *J. Photochem. Photobiol B: Biol.* **183**, 266–274 (2018). 10.1016/j.jphotobiol.2018.04.039 29747146

[71] Merchant, S. H., Gonchoroff, N. J. & Hutchison, R. E. Apoptotic index by Annexin V flow cytometry: adjunct to morphologic and cytogenetic diagnosis of myelodysplastic syndromes. *Cytometry: J. Int. Soc. Anal. Cytol.* **46**, 28–32 (2001). 10.1002/1097-0320(20010215)46:1<28::aid-cyto1034>3.0.co;2-h 11241504

[72] Potten, C. S. What is an apoptotic index measuring? A commentary. *Br. J. Cancer*. **74**, 1743 (1996). 8956787 10.1038/bjc.1996.624 PMC2077230

[73] Livak, K. J. & Schmittgen, T. D. Analysis of relative gene expression data using real-time quantitative PCR and the 2(-Delta Delta C(T)) Method. *Methods* 25, 402–408, (2001). 10.1006/meth.2001.1262 10.1006/meth.2001.1262 11846609

[74] Khodapasand, E., Jafarzadeh, N., Farrokhi, F., Kamalidehghan, B. & Houshmand, M. Is Bax/Bcl-2 ratio considered as a prognostic marker with age and tumor location in colorectal cancer? *Iran. Biomed. J.* **19**, 69 (2015). 25864810 10.6091/ibj.1366.2015 PMC4412916

[75] Matsumoto, H. et al. Bax to Bcl-2 ratio and Ki-67 index are useful predictors of neoadjuvant chemoradiation therapy in bladder cancer. *Jpn J. Clin. Oncol.* **34**, 124–130 (2004). 15078907 10.1093/jjco/hyh026

[76] Raisova, M. et al. The Bax/Bcl-2 ratio determines the susceptibility of human melanoma cells to CD95/Fas-mediated apoptosis. *J. Invest. Dermatology*. **117**, 333–340 (2001). 10.1046/j.0022-202x.2001.01409.x 11511312

[77] Zhu, L. et al. Curcumin triggers apoptosis via upregulation of Bax/Bcl-2 ratio and caspase activation in SW872 human adipocytes. *Mol. Med. Rep.* **12**, 1151–1156 (2015). 25760477 10.3892/mmr.2015.3450

[78] Sharifi, S., Barar, J., Hejazi, M. S. & Samadi, N. Roles of the Bcl-2/Bax ratio, caspase-8 and 9 in resistance of breast cancer cells to Paclitaxel. *Asian Pac. J. Cancer Prev.* **15**, 8617–8622 (2014). 25374178 10.7314/apjcp.2014.15.20.8617

[79] Burton, K. A study of the conditions and mechanism of the diphenylamine reaction for the colorimetric Estimation of deoxyribonucleic acid. *Biochem. J.* **62**, 315–323. 10.1042/bj0620315 (1956). 13293190 10.1042/bj0620315 PMC1215910

[80] Gibb, R. K. & Gercel-Taylor, C. Use of diphenylamine in the detection of apoptosis. *Methods Mol. Med.* **39**, 679–680. 10.1385/1-59259-071-3:679 (2001). 21340829 10.1385/1-59259-071-3:679

[81] Gibb, R. K. et al. Apoptosis as a measure of chemosensitivity to cisplatin and taxol therapy in ovarian cancer cell lines. *Gynecol. Oncol.* **65**, 13–22. 10.1006/gyno.1997.4637 (1997). 9103385 10.1006/gyno.1997.4637

[82] Grada, A., Otero-Vinas, M., Prieto-Castrillo, F., Obagi, Z. & Falanga, V. Research techniques made simple: analysis of collective cell migration using the wound healing assay. *J. Invest. Dermatol.* **137**, e11–e16 (2017). 28110712 10.1016/j.jid.2016.11.020

[83] Huang, H. L. et al. Anticancer activity of MPT0E028, a novel potent histone deacetylase inhibitor, in human colorectal cancer HCT116 cells in vitro and in vivo. (2012). 10.1371/journal.pone.0043645 PMC3425516 22928010

[84] Gudneppanavar, R. et al. Epigenetic histone modification by butyrate downregulates KIT and attenuates mast cell function. *J. Cell. Mol. Med.* **27**, 2983–2994 (2023). 37603611 10.1111/jcmm.17924 PMC10538265

[85] Wu, H., Van Der Pol, W. J., Dubois, L. G., Morrow, C. D. & Tollefsbol, T. O. Dietary supplementation of inulin contributes to the prevention of Estrogen Receptor-Negative mammary Cancer by alteration of gut microbial communities and epigenetic regulations. *Int. J. Mol. Sci.* **24**, 9015 (2023). 37240357 10.3390/ijms24109015 PMC10218871

[86] An, T. J. et al. Tiotropium bromide improves neutrophilic asthma by recovering histone deacetylase 2 activity. *J. Korean Med. Sci.* **38** (2023). 10.3346/jkms.2023.38.e91 PMC10042725 36974400

[87] Arora, I., Li, S., Crowley, M. R., Li, Y. & Tollefsbol, T. O. Genome-wide analysis on transcriptome and methylome in prevention of mammary tumor induced by early life combined botanicals. *Cells* **12**, 14 (2022). 36611809 10.3390/cells12010014 PMC9818885

[88] Rueff, J. & Rodrigues, A. S. Cancer drug resistance: A brief overview from a genetic viewpoint. *Cancer Drug Resistance: Overviews Methods*, 1–18 (2016). 10.1007/978-1-4939-3347-1_1 26910065

[89] Ashique, S. et al. Multi drug resistance in colorectal Cancer-approaches to overcome, advancements and future success. *Adv. Cancer Biology-Metastasis*. **10**, 100114 (2024).

[90] Li, Q. et al. Signaling pathways involved in colorectal cancer: pathogenesis and targeted therapy. *Signal. Transduct. Target. Therapy*. **9**, 266 (2024). 10.1038/s41392-024-01953-7 PMC11456611 39370455

[91] Smith, A. J. & Hergenrother, P. J. Raptinal: a powerful tool for rapid induction of apoptotic cell death. *Cell. Death Discovery*. **10**, 371 (2024). 39164225 10.1038/s41420-024-02120-1 PMC11335860

[92] Taha, H., Elfar, N., Haffez, H. & Hassan, Z. A. Raptinal silver nanoparticles: new therapeutic advances in hepatocellular carcinoma mouse model. *Naunyn-Schmiedeberg’s Arch. Pharmacol.* **394**, 279–289. 10.1007/s00210-020-01973-4 (2021). 32945892 10.1007/s00210-020-01973-4

[93] Feitelson, M. A. et al. in *Semin. Cancer Biol.* S25-S54 (Elsevier).

[94] Sawasdee, N. et al. Doxorubicin sensitizes breast cancer cells to natural killer cells in connection with increased Fas receptors. *Int. J. Mol. Med.* **49**, 40 (2022). 35119077 10.3892/ijmm.2022.5095 PMC8815410

[95] Guardamagna, I. et al. An integrated analysis of the response of colorectal adenocarcinoma Caco-2 cells to X-Ray exposure. *Front. Oncol.* **11**, 688919 (2021). 34150657 10.3389/fonc.2021.688919 PMC8209426

[96] Lo, Y. L. & Liu, Y. Reversing multidrug resistance in Caco-2 by Silencing MDR1, MRP1, MRP2, and BCL-2/BCL-xL using liposomal antisense oligonucleotides. *PLoS One*. **9**, e90180 (2014). 24637737 10.1371/journal.pone.0090180 PMC3956467

[97] Qureshi-Baig, K., Ullmann, P., Haan, S. & Letellier, E. T umor-I nitiating C Ells: a cri TIC al review of isolation approaches and new challenges in targeting strategies. *Mol. Cancer*. **16**, 1–16 (2017). 28209178 10.1186/s12943-017-0602-2 PMC5314476

[98] Dobrotkova, V., Chlapek, P., Mazanek, P., Sterba, J. & Veselska, R. Traffic lights for retinoids in oncology: molecular markers of retinoid resistance and sensitivity and their use in the management of cancer differentiation therapy. *BMC Cancer*. **18**, 1–13 (2018). 30384831 10.1186/s12885-018-4966-5 PMC6211450

[99] Shilkaitis, A., Green, A. & Christov, K. Retinoids induce cellular senescence in breast cancer cells by RAR-β dependent and independent pathways: potential clinical implications. *Int. J. Oncol.* **47**, 35–42 (2015). 25997921 10.3892/ijo.2015.3013 PMC4485653

[100] Ling, Y. H., El-Naggar, A. K., Priebe, W. & Perez-Soler, R. Cell cycle-dependent cytotoxicity, G2/M phase arrest, and disruption of p34cdc2/cyclin B1 activity induced by doxorubicin in synchronized P388 cells. *Mol. Pharmacol.* **49**, 832–841 (1996). 8622633

[101] Kim, H. S., Lee, Y. S. & Kim, D. K. Doxorubicin exerts cytotoxic effects through cell cycle arrest and Fas-mediated cell death. *Pharmacology* **84**, 300–309 (2009). 19829019 10.1159/000245937

[102] Newell, M., Baker, K., Postovit, L. M. & Field, C. J. A critical review on the effect of docosahexaenoic acid (DHA) on cancer cell cycle progression. *Int. J. Mol. Sci.* **18**, 1784 (2017). 28817068 10.3390/ijms18081784 PMC5578173

[103] Chakraborty, S. P. et al. Internalization of Staphylococcus aureus in lymphocytes induces oxidative stress and DNA fragmentation: possible ameliorative role of nanoconjugated vancomycin. *Oxid. Med. Cell. Longev.* (2011). (2011). 10.1155/2011/942123 PMC3175730 21941607

[104] Salam, S. G. A. et al. Cell growth Inhibition, DNA fragmentation and Apoptosis-Inducing properties of Household-Processed leaves and seeds of Fenugreek (Trigonella Foenum-Graecum Linn.) against HepG2, HCT-116, and MCF-7 cancerous cell lines. *Curr. Issues Mol. Biol.* **45**, 936–953 (2023). 36826005 10.3390/cimb45020060 PMC9955320

[105] Higuchi, Y. & Chromosomal DNA fragmentation in apoptosis and necrosis induced by oxidative stress. *Biochem. Pharmacol.* **66**, 1527–1535 (2003). 14555231 10.1016/s0006-2952(03)00508-2

[106] Amalina, N. D., Salsabila, I. A., Zulfin, U. M., Jenie, R. I. & Meiyanto, E. In vitro synergistic effect of hesperidin and doxorubicin downregulates epithelial-mesenchymal transition in highly metastatic breast cancer cells. *J. Egypt. Natl. Canc Inst.* **35**, 1–13 (2023). 36967442 10.1186/s43046-023-00166-3 PMC13313971

[107] Alfarouk, K. O. et al. Resistance to cancer chemotherapy: failure in drug response from ADME to P-gp. *Cancer Cell. Int.* **15**, 1–13 (2015). 26180516 10.1186/s12935-015-0221-1 PMC4502609

[108] Lei, Z. N. et al. Understanding and targeting resistance mechanisms in cancer. *MedComm* **4**, e265 (2023). 37229486 10.1002/mco2.265 PMC10203373

[109] Priestman, T. & Priestman, T. Some practical aspects of cancer chemotherapy. *Cancer Chemother. Clin. Pract.*, 35–77 (2008).

[110] Handling, S. 18 AntineoplAstic therApy. *Infusion Nursing: An Evidence-Based Approach*, 351 (2009).

[111] Cosh, A. & Carslaw, H. What does a GP need to know about chemotherapy? *InnovAiT* **6**, 197–205 (2013).

[112] Kummar, S. et al. Utilizing targeted cancer therapeutic agents in combination: novel approaches and urgent requirements. *Nat. Rev. Drug Discovery*. **9**, 843–856 (2010). 21031001 10.1038/nrd3216

[113] Duarte, D. & Vale, N. Evaluation of synergism in drug combinations and reference models for future orientations in oncology. *Curr. Res. Pharmacol. Drug Discovery*. **3**, 100110. 10.1016/j.crphar.2022.100110 (2022). 10.1016/j.crphar.2022.100110 PMC9127325 35620200

[114] Yang, C. et al. Natural products in preventing tumor drug resistance and related signaling pathways. *Molecules* **27**, 3513 (2022). 35684449 10.3390/molecules27113513 PMC9181879

[115] Jaaks, P. et al. Effective drug combinations in breast, colon and pancreatic cancer cells. *Nature* **603**, 166–173 (2022). 35197630 10.1038/s41586-022-04437-2 PMC8891012

[116] Thorn, C. F. et al. Doxorubicin pathways: pharmacodynamics and adverse effects. *Pharmacogenet Genomics*. **21**, 440 (2011). 21048526 10.1097/FPC.0b013e32833ffb56 PMC3116111

[117] Mehdizadeh, K., Ataei, F. & Hosseinkhani, S. Effects of doxorubicin and docetaxel on susceptibility to apoptosis in high expression level of survivin in HEK and HEK-S cell lines as in vitro models. *Biochem. Biophys. Res. Commun.* **532**, 139–144 (2020). 32828533 10.1016/j.bbrc.2020.08.028

[118] Abd-Rabou, A. A., Abdelaziz, A. M., Shaker, O. G. & Ayeldeen, G. Hyaluronated nanoparticles deliver raloxifene to CD44-expressed colon cancer cells and regulate LncRNAs/miRNAs epigenetic cascade. *Cancer Nanotechnol*. **14**, 1–23 (2023).

[119] Tsujii, M., Kawano, S. & DuBois, R. N. Cyclooxygenase-2 expression in human colon cancer cells increases metastatic potential. *Proceedings of the National Academy of Sciences* 94, 3336–3340 (1997). 10.1073/pnas.94.7.3336 PMC20370 9096394

[120] Zeya, B. et al. Diosmin in combination with naringenin enhances apoptosis in colon cancer cells. *Oncol. Rep.* **47**, 1–11 (2022). 34738632 10.3892/or.2021.8215

[121] Sonowal, H. et al. Aldose reductase inhibitor increases doxorubicin-sensitivity of colon cancer cells and decreases cardiotoxicity. *Sci. Rep.* **7**, 3182 (2017). 28600556 10.1038/s41598-017-03284-w PMC5466629

[122] Effat, H., Abosharaf, H. A. & Radwan, A. M. Combined effects of naringin and doxorubicin on the JAK/STAT signaling pathway reduce the development and spread of breast cancer cells. *Sci. Rep.* **14**, 2824 (2024). 38310190 10.1038/s41598-024-53320-9 PMC10838285

[123] Lin, K. et al. Combination of ruthenium complex and doxorubicin synergistically inhibits cancer cell growth by down-regulating PI3K/AKT signaling pathway. *Front. Oncol.* **10**, 141 (2020). 32133289 10.3389/fonc.2020.00141 PMC7041628

[124] Tong, N. et al. Berberine sensitizes Mutliple human cancer cells to the anticancer effects of doxorubicin in vitro. *Oncol. Lett.* **3**, 1263–1267 (2012). 22783430 10.3892/ol.2012.644 PMC3392583

[125] Suski, J. M., Braun, M., Strmiska, V. & Sicinski, P. Targeting cell-cycle machinery in cancer. *Cancer Cell.* **39**, 759–778 (2021). 33891890 10.1016/j.ccell.2021.03.010 PMC8206013

[126] Otto, T. & Sicinski, P. Cell cycle proteins as promising targets in cancer therapy. *Nat. Rev. Cancer*. **17**, 93–115 (2017). 28127048 10.1038/nrc.2016.138 PMC5345933

[127] Fan, C. et al. Strategy to enhance the therapeutic effect of doxorubicin in human hepatocellular carcinoma by Selenocystine, a synergistic agent that regulates the ROS-mediated signaling. *Oncotarget* **5**, 2853 (2014). 24797310 10.18632/oncotarget.1854 PMC4058050

[128] Kari, S. et al. Programmed cell death detection methods: a systematic review and a categorical comparison. *Apoptosis* **27**, 482–508 (2022). 35713779 10.1007/s10495-022-01735-y PMC9308588

[129] Mohammad, R. M. et al. in *Semin. Cancer Biol.* S78-S103 (Elsevier).

[130] Stephens, A. D. et al. Chromatin histone modifications and rigidity affect nuclear morphology independent of lamins. *Mol. Biol. Cell.* **29**, 220–233 (2018). 29142071 10.1091/mbc.E17-06-0410 PMC5909933

[131] Hseu, Y. C. et al. Flavokawain B and doxorubicin work synergistically to impede the propagation of gastric cancer cells via ROS-mediated apoptosis and autophagy pathways. *Cancers (Basel)*. **12**, 2475 (2020). 32882870 10.3390/cancers12092475 PMC7564097

[132] Efferth, T., Giaisi, M., Merling, A., Krammer, P. H. & Li-Weber, M. Artesunate induces ROS-mediated apoptosis in doxorubicin-resistant T leukemia cells. *PLoS One*. **2**, e693 (2007). 17668070 10.1371/journal.pone.0000693 PMC1933253

[133] Mirgorodskaya, A. et al. Supramolecular tools to improve wound healing and antioxidant properties of abietic acid: biocompatible microemulsions and emulgels. *Molecules* **27**10.3390/molecules27196447 (2022). 10.3390/molecules27196447 PMC9572722 36234983

[134] Wahab, N. A. A., Giribabu, N., Kilari, E. K. & Salleh, N. Abietic acid ameliorates nephropathy progression via mitigating renal oxidative stress, inflammation, fibrosis and apoptosis in high fat diet and low dose streptozotocin-induced diabetic rats. *Phytomedicine* **107**, 154464. 10.1016/j.phymed.2022.154464 (2022). 36215789 10.1016/j.phymed.2022.154464

[135] Gogul Ramnath, M., Thirugnanasampandan, R., Sadasivam, M., Subramaniam Mohan, P. & Antioxidant Antibacterial and antiacetylcholinesterase activities of abietic acid from Isodon Wightii (Bentham) H. Hara. *Free Radicals Antioxid.* **5**, 01–05. 10.5530/fra.2015.1.1 (2015).

[136] Fernández, M. A. et al. Anti-inflammatory activity of abietic acid, a diterpene isolated from Pimenta racemosa Var. Grissea. *J. Pharm. Pharmacol.* **53**, 867–872. 10.1211/0022357011776027 (2001). 11428663 10.1211/0022357011776027

[137] Aboyewa, J. A. et al. Co-treatment of Caco-2 cells with doxorubicin and gold nanoparticles produced from cyclopia intermedia extracts or mangiferin enhances drug effects. *Nanomaterials* **12**, 3918 (2022). 36364694 10.3390/nano12213918 PMC9654788

[138] Norouzi, M. et al. Doxorubicin-loaded iron oxide nanoparticles for glioblastoma therapy: A combinational approach for enhanced delivery of nanoparticles. *Sci. Rep.* **10**, 11292 (2020). 32647151 10.1038/s41598-020-68017-y PMC7347880

[139] Wang, N. & Feng, Y. Elaborating the role of natural products-induced autophagy in cancer treatment: achievements and artifacts in the state of the art. *BioMed research international* 934207 (2015). (2015). 10.1155/2015/934207 PMC4363717 25821829

[140] Oncel, S. et al. Efficacy of butyrate to inhibit colonic Cancer cell growth is cell Type-Specific and Apoptosis-Dependent. *Nutrients* **16**, 529 (2024). 38398853 10.3390/nu16040529 PMC10892417

[141] Untereiner, A. A. et al. Drug resistance induces the upregulation of H2S-producing enzymes in HCT116 colon cancer cells. *Biochem. Pharmacol.* **149**, 174–185 (2018). 29061341 10.1016/j.bcp.2017.10.007 PMC5866167

[142] De Angelis, P. M. et al. Molecular characterizations of derivatives of HCT116 colorectal cancer cells that are resistant to the chemotherapeutic agent 5-fluorouracil. *Int. J. Oncol.* **24**, 1279–1288 (2004). 15067352

[143] Gao, J., Hou, D., Hu, P. & Mao, G. Curcumol increases the sensitivity of colon cancer to 5-FU by regulating Wnt/β-catenin signaling. *Translational Cancer Res.* **10**, 2437 (2021). 10.21037/tcr-21-689 PMC8798486 35116559

[144] Coronel-Hernández, J. et al. Combination of Metformin, sodium oxamate and doxorubicin induces apoptosis and autophagy in colorectal cancer cells via downregulation HIF-1α. *Front. Oncol.* **11**, 594200 (2021). 34123772 10.3389/fonc.2021.594200 PMC8187873

[145] Wani, A. K. et al. Targeting apoptotic pathway of cancer cells with phytochemicals and plant-based nanomaterials. *Biomolecules* **13**, 194 (2023). 36830564 10.3390/biom13020194 PMC9953589

[146] Wolf, P., Schoeniger, A. & Edlich, F. Pro-apoptotic complexes of BAX and BAK on the outer mitochondrial membrane. *Biochim. Et Biophys. Acta (BBA)-Molecular Cell. Res.* **1869**, 119317 (2022). 10.1016/j.bbamcr.2022.119317 35752202

[147] Karch, J. et al. Bax and bak function as the outer membrane component of the mitochondrial permeability pore in regulating necrotic cell death in mice. *Elife* **2**, e00772 (2013). 23991283 10.7554/eLife.00772 PMC3755340

[148] Vaseva, A. V. et al. p53 opens the mitochondrial permeability transition pore to trigger necrosis. *Cell* **149**, 1536–1548 (2012). 22726440 10.1016/j.cell.2012.05.014 PMC3383624

[149] Murphy, M. E., Leu, J. I. J. & George, D. L. p53 moves to mitochondria: a turn on the path to apoptosis. *Cell. Cycle*. **3**, 834–837 (2004). 15190209

[150] Eleftheriadis, T., Pissas, G., Liakopoulos, V. & Stefanidis, I. Cytochrome C as a potentially clinical useful marker of mitochondrial and cellular damage. *Front. Immunol.* **7**, 211196 (2016). 10.3389/fimmu.2016.00279 PMC4951490 27489552

[151] Kirkinezos, I. G. et al. Cytochrome C association with the inner mitochondrial membrane is impaired in the CNS of G93A-SOD1 mice. *J. Neurosci.* **25**, 164–172 (2005). 15634778 10.1523/JNEUROSCI.3829-04.2005 PMC6725219

[152] Boland, K., Flanagan, L. & Prehn, J. H. Paracrine control of tissue regeneration and cell proliferation by Caspase-3. *Cell. Death Dis.* **4**, e725–e725 (2013). 23846227 10.1038/cddis.2013.250 PMC3730423

[153] D’amelio, M., Cavallucci, V. & Cecconi, F. Neuronal caspase-3 signaling: not only cell death. *Cell. Death Differ.* **17**, 1104–1114 (2010). 19960023 10.1038/cdd.2009.180

[154] Jiang, H., Zhao, P. J., Su, D., Feng, J. & Ma, S. L. Paris saponin I induces apoptosis via increasing the Bax/Bcl–2 ratio and caspase–3 expression in gefitinib–resistant non–small cell lung cancer in vitro and in vivo. *Mol. Med. Rep.* **9**, 2265–2272 (2014). 24718383 10.3892/mmr.2014.2108

[155] Gazzaniga, P. et al. bcl-2/bax mRNA expression ratio as prognostic factor in low‐grade urinary bladder cancer. *Int. J. Cancer*. **69**, 100–104 (1996). 8608975 10.1002/(SICI)1097-0215(19960422)69:2<100::AID-IJC5>3.0.CO;2-4

[156] Takahashi, M., Mukai, H., Toshimori, M., Miyamoto, M. & Ono, Y. Proteolytic activation of PKN by caspase-3 or related protease during apoptosis. *Proc. Natl. Acad. Sci.* **95**, 11566–11571 (1998). 9751706 10.1073/pnas.95.20.11566 PMC21681

[157] Liu, P. F. et al. Expression levels of cleaved caspase-3 and caspase-3 in tumorigenesis and prognosis of oral tongue squamous cell carcinoma. *PLoS One*. **12**, e0180620 (2017). 28700659 10.1371/journal.pone.0180620 PMC5503265

[158] Howard, G. R., Jost, T. A., Yankeelov, T. E. & Brock, A. Quantification of long-term doxorubicin response dynamics in breast cancer cell lines to direct treatment schedules. *PLoS Comput. Biol.* **18**, e1009104. 10.1371/journal.pcbi.1009104 (2022). 35358172 10.1371/journal.pcbi.1009104 PMC9004764

[159] Aboyewa, J. A. et al. Co-Treatment of Caco-2 cells with doxorubicin and gold nanoparticles produced from cyclopia intermedia extracts or mangiferin enhances drug effects. *Nanomaterials (Basel Switzerland)*. **12**10.3390/nano12213918 (2022). 10.3390/nano12213918 PMC9654788 36364694

[160] Hu, T., Li, Z., Gao, C. Y. & Cho, C. H. Mechanisms of drug resistance in colon cancer and its therapeutic strategies. *World J. Gastroenterol.* **22**, 6876–6889. 10.3748/wjg.v22.i30.6876 (2016). 27570424 10.3748/wjg.v22.i30.6876 PMC4974586

[161] Rushing, B. R., Molina, S. & Sumner, S. Metabolomics analysis reveals altered metabolic pathways and response to doxorubicin in Drug-Resistant Triple-Negative breast Cancer cells. *Metabolites* **13**, 865 (2023). 37512572 10.3390/metabo13070865 PMC10383792

[162] Hunter, J., Hirst, B. H. & Simmons, N. L. Drug absorption limited by P-glycoprotein-mediated secretory drug transport in human intestinal epithelial Caco-2 cell layers. *Pharm. Res.* **10**, 743–749. 10.1023/a:1018972102702 (1993). 8100632 10.1023/a:1018972102702

[163] Taipalensuu, J. et al. Correlation of gene expression of ten drug efflux proteins of the ATP-binding cassette transporter family in normal human jejunum and in human intestinal epithelial Caco-2 cell monolayers. *J. Pharmacol. Exp. Ther.* **299**, 164–170 (2001). 11561076

[164] Guo, Y. et al. Sinapine as an active compound for inhibiting the proliferation of Caco-2 cells via downregulation of P-glycoprotein. *Food Chem. Toxicol.* **67**, 187–192. 10.1016/j.fct.2014.02.035 (2014). 24607798 10.1016/j.fct.2014.02.035

[165] Kciuk, M. et al. Doxorubicin-An agent with multiple mechanisms of anticancer activity. *Cells* **12**10.3390/cells12040659 (2023). 10.3390/cells12040659 PMC9954613 36831326

[166] Wang, S. et al. Doxorubicin induces apoptosis in normal and tumor cells via distinctly different mechanisms. Intermediacy of H(2)O(2)- and p53-dependent pathways. *J. Biol. Chem.* **279**, 25535–25543. 10.1074/jbc.M400944200 (2004). 15054096 10.1074/jbc.M400944200

[167] Aboyewa, J. A. et al. Co-Treatment of Caco-2 Cells with Doxorubicin and Gold Nanoparticles Produced from Cyclopia intermedia Extracts or Mangiferin Enhances Drug Effects. *Nanomaterials* 12, 3918 (2022). 10.3390/nano12213918 PMC9654788 36364694

[168] Roberts, N. J., Zhou, S., Diaz Jr, L. A. & Holdhoff, M. Systemic use of tumor necrosis factor alpha as an anticancer agent. *Oncotarget* **2**, 739 (2011). 22036896 10.18632/oncotarget.344 PMC3248159

[169] Wong, V. K. W. et al. Saikosaponin-d enhances the anticancer potency of TNF-via overcoming its undesirable response of activating NF-Kappa B signalling in cancer cells. *Evid. Based Complement. Alternat. Med.* (2013). (2013). 10.1155/2013/745295 PMC3610377 23573150

[170] Aggarwal, B. B., Gupta, S. C. & Kim, J. H. Historical perspectives on tumor necrosis factor and its superfamily: 25 years later, a golden journey. *Blood J. Am. Soc. Hematol.* **119**, 651–665 (2012). 10.1182/blood-2011-04-325225 PMC3265196 22053109

[171] Balkwill, F. Tumour necrosis factor and cancer. *Nat. Rev. Cancer*. **9**, 361–371 (2009). 19343034 10.1038/nrc2628

[172] Naimi, A. et al. TNF-related apoptosis-inducing ligand (TRAIL) as the potential therapeutic target in hematological malignancies. *Biomed. Pharmacother*. **98**, 566–576 (2018). 29288972 10.1016/j.biopha.2017.12.082

[173] Nelson, V. K. et al. Reactive oxygen species mediated apoptotic death of colon cancer cells: therapeutic potential of plant derived alkaloids. *Front. Endocrinol. (Lausanne)*. **14**, 1201198 (2023). 37560308 10.3389/fendo.2023.1201198 PMC10408138

[174] Kim, J., Lee, S., Park, J. & Yoo, Y. TNF-α-induced ROS production triggering apoptosis is directly linked to Romo1 and Bcl-XL. *Cell. Death Differ.* **17**, 1420–1434 (2010). 20203691 10.1038/cdd.2010.19

[175] Hsu, H., Shu, H. B., Pan, M. G. & Goeddel, D. V. TRADD–TRAF2 and TRADD–FADD interactions define two distinct TNF receptor 1 signal transduction pathways. *Cell* **84**, 299–308 (1996). 8565075 10.1016/s0092-8674(00)80984-8

[176] Hsu, H., Xiong, J. & Goeddel, D. V. The TNF receptor 1-associated protein TRADD signals cell death and NF-κB activation. *Cell* **81**, 495–504 (1995). 7758105 10.1016/0092-8674(95)90070-5

[177] Ververis, K. & Karagiannis, T. C. An atlas of histone deacetylase expression in breast cancer: fluorescence methodology for comparative semi-quantitative analysis. *Am. J. Translational Res.* **4**, 24 (2012). PMC3276375 22347520

[178] Sudo, T. et al. Histone deacetylase 1 expression in gastric cancer. *Oncol. Rep.* **26**, 777–782 (2011). 21725604 10.3892/or.2011.1361

[179] You, B. R., Han, B. R. & Park, W. H. Suberoylanilide hydroxamic acid increases anti-cancer effect of tumor necrosis factor-α through up-regulation of TNF receptor 1 in lung cancer cells. *Oncotarget* **8**, 17726 (2017). 28099148 10.18632/oncotarget.14628 PMC5392281

[180] Filip, S. et al. Distant metastasis in colorectal cancer patients—do we have new predicting clinicopathological and molecular biomarkers? A comprehensive review. *Int. J. Mol. Sci.* **21**, 5255 (2020). 32722130 10.3390/ijms21155255 PMC7432613

[181] Pan, D. et al. Oxymatrine synergistically enhances doxorubicin anticancer effects in colorectal cancer. *Front. Pharmacol.* **12**, 673432 (2021). 34305593 10.3389/fphar.2021.673432 PMC8297828

[182] Wang, X., Decker, C. C., Zechner, L., Krstin, S. & Wink, M. In vitro wound healing of tumor cells: Inhibition of cell migration by selected cytotoxic alkaloids. *BMC Pharmacol. Toxicol.* **20**, 1–12 (2019). 30626448 10.1186/s40360-018-0284-4 PMC6327619

