# Supplementary material for: Synergistic effects of abietic acid combined with doxorubicin on apoptosis induction in a human colorectal cancer cell line
Source: Sci Rep. 2025 May 8;15:16102. doi: 10.1038/s41598-025-99616-2 (PMC12062260; doi:10.1038/s41598-025-99616-2)
Supplement: Supplementary file 1 — Supplementary Material 1 [file 41598_2025_99616_MOESM1_ESM.docx]

**Synergistic effects of abietic acid combined with doxorubicin on apoptosis induction in a human colorectal cancer cell line**

**Table 4S**: Apoptosis assay analysis of the percentage of the HCT-116 cell subpopulation and distribution of cells in the various apoptosis stages as measured by flow cytometry. The IC_50_s of abietic acid, doxorubicin or their combination were applied to HCT-116 cells, and the results were compared to those obtained with 0.1% DMSO as the negative control. The combination of doxorubicin and doxorubicin dramatically reduced the percentage of viable cells through the induction of late apoptosis and necrosis. The data are expressed as the mean ± SEM; n = 3. 2D apoptosis plot was divided into four quadrant (Lower left (LL), Lower right (LR), Upper left (UL), Upper right (UR)).

| **Tested compounds** | **% Q1-LL** | **% Q2-LR** | **% Q3-UL** | **% Q4-UR** |
| --- | --- | --- | --- | --- |
| **Negative control** | 88.89 ± 10.6 | 7.18 ± 1.5 | 1.70 ± 0.2 | 2.23 ± 0.8 |
| **Abietic** | 73.75 ± 11.7 | 4.05 ± 1.1 | 14.87 ± 1.4^***^ | 7.34 ± 3.3^*^ |
| **Doxorubicin** | 59.46 ± 9.4^*^ | 8.09 ± 2.1 | 9.80 ± 2.7^***^ | 22.65 ± 5.7^***^ |
| **Abietic acid-Doxorubicin combination** | 33.38 ± 7.8^**^ | 0.83 ± 0.1 | 43.07 ± 9.8^***^ | 22.72 ± 3.1^***^ |

*^*^ P<0.05, ^**^ P<0.01, ^***^ P<0.001*
