# Supplementary material for: Synergistic effects of abietic acid combined with doxorubicin on apoptosis induction in a human colorectal cancer cell line
Source: Sci Rep. 2025 May 8;15:16102. doi: 10.1038/s41598-025-99616-2 (PMC12062260; doi:10.1038/s41598-025-99616-2)
Supplement: Supplementary file 2 — Supplementary Material 2 [file 41598_2025_99616_MOESM2_ESM.docx]

**Synergistic effects of abietic acid combined with doxorubicin on apoptosis induction in a human colorectal cancer cell line**

**Table 1S**: Primer’s sequence of key modulatory genes used for gene expression analysis.

| **Gene** | **Forward primer sequence** | **reverse primer sequence** |
| --- | --- | --- |
| Caspase-3 | 5'- ACATGGAAGCGAATCAATGGACTC -3' | 5'- AAGGACTCAAATTCTGTTGCCACC -3' |
| Cyt-c | 5'- GAGGCAAGCATAAGACTGGA -3' | 5'- TACTCCATCAGGGTATCCTC -3' |
| Bax | 5'- CCCGAGAGGTCTTTTTCCGAG -3' | 5'- CCAGCCCATGATGGTTCTGAT -3' |
| Bcl-2 | 5'- TTGTGGCCTTCTTTGAGTTCGGTG -3' | 5'- GGTGCCGGTTCAGGTACTCAGTCA -3' |
| p53 | 5'- GCCCAACAACACCAGCTCCT -3' | 5'- CCTGGGCATCCTTGAGTTCC -3' |
| NFkB | 5'- ATGGCTTCTATGAGGCTGAG -3' | 5'- GTTGTTGTTGGTCTGGATGC -3' |
| TNF-α | 5'- ATGAGCACTGAAAGCATGATCC -3' | 5'- GAGGGCTGATTAGAGAGAGGTC -3' |
| GAPDH | 5'- CTGACTTCAACAGCGACACC -3' | 5'- TAGCCAAATTCGTTGTCATACC -3' |
