# Supplementary material for: Synergistic effects of abietic acid combined with doxorubicin on apoptosis induction in a human colorectal cancer cell line
Source: Sci Rep. 2025 May 8;15:16102. doi: 10.1038/s41598-025-99616-2 (PMC12062260; doi:10.1038/s41598-025-99616-2)
Supplement: Supplementary file 3 — Supplementary Material 3 [file 41598_2025_99616_MOESM3_ESM.docx]

**Synergistic effects of abietic acid combined with doxorubicin on apoptosis induction in a human colorectal cancer cell line**

**Table 5S**: Cell cycle analysis of the percentage of the HCT-116 cell subpopulation and distribution of cells in the various cell cycle stages as measured by flow cytometry. The IC_50_s of abietic acid, doxorubicin or their combination were applied to HCT-116 cells, and the results were compared to those obtained with 0.1% DMSO as the negative control. Doxorubicin and its combination can cause dramatic cell cycle arrest at various stages of the cell cycle. The data are expressed as the mean ± SEM; n = 3.

| **Tested compounds** | **% SubG_0_-G_1_** | **% G_0_-G_1_** | **% S** | **% G_2_M** |
| --- | --- | --- | --- | --- |
| **Negative control** | 2.18 ± 0.6 | 66.13 ± 11.4 | 11.04 ± 2.6 | 20.54 ± 5.3 |
| **Abietic** | 26.88 ± 4.3^**^ | 53.91 ± 6.5 | 6.17 ± 1.3 | 11.88 ± 2.7 |
| **Doxorubicin** | 46.71 ± 10.4^***^ | 37.10 ± 8.5^*^ | 8.85 ± 1.8 | 6.11 ± 3.2^*^ |
| **Abietic acid-Doxorubicin combination** | 48.03 ± 11.6^***^ | 38.20 ± 6.4^*^ | 6.00 ± 1.1 | 6.33 ± 2.4^*^ |

*^*^ P<0.05, ^**^ P<0.01, ^***^ P<0.001*
