# Supplementary material for: Synergistic effects of abietic acid combined with doxorubicin on apoptosis induction in a human colorectal cancer cell line
Source: Sci Rep. 2025 May 8;15:16102. doi: 10.1038/s41598-025-99616-2 (PMC12062260; doi:10.1038/s41598-025-99616-2)
Supplement: Supplementary file 4 — Supplementary Material 4 [file 41598_2025_99616_MOESM4_ESM.docx]

**Synergistic effects of abietic acid combined with doxorubicin on apoptosis induction in a human colorectal cancer cell line**

**Table 2S**: Effect of the combined IC_50_ as individual compounds (abietic acid and doxorubicin) and in combination in Caco-2 cells. There was a noticeable proliferative effect on normal cells with all the doses suggesting minimal cytotoxic effect on normal cells. The data are presented as the means ± SEMs of n = 3.

| **Tested compounds** | **Percentage Growth** | **Percentage growth inhibition (GI%)** |
| --- | --- | --- |
| Negative control | 100% | 0% |
| IC_50_ Abietic (in combination) | 123.8% | 0% |
| IC_50_ Abietic (in combination) | 115.1% | 0% |
| IC_50_ Abietic (in combination) | 112.9% | 0% |
