# Supplementary material for: Synergistic effects of abietic acid combined with doxorubicin on apoptosis induction in a human colorectal cancer cell line
Source: Sci Rep. 2025 May 8;15:16102. doi: 10.1038/s41598-025-99616-2 (PMC12062260; doi:10.1038/s41598-025-99616-2)
Supplement: Supplementary file 5 — Supplementary Material 5 [file 41598_2025_99616_MOESM5_ESM.docx]

**Synergistic effects of abietic acid combined with doxorubicin on apoptosis induction in a human colorectal cancer cell line**

**Table 3S**: Combinations of individual compounds (abietic acid and doxorubicin) in HCT-116 cells using fixed ratios of the individual IC_50_ values. There was a noticeable synergistic effect when using the isobologram technique. The synergistic impact was confirmed by the combination index (CI) and dose reduction index (DRI). The data are presented as the means ± SEMs of n = 3.

| **Tested compounds** | **IC_50_ (µM)** | **×IC_50_ (Combination)**  **(µM)** | **IC_50_ (in combination)**  **(µM)** | **DRI** | **CI** |
| --- | --- | --- | --- | --- | --- |
| Doxorubicin | 17.53 ± 5.3 | ------- | 6.8 ± 1.6 | ------- | ------- |
| Abietic acid | 13.73 ± 1.9 | 0.388 ± 0.02 | 5.3 ± 0.9 | 4.0 | 0.63 |
