# Supplementary material for: Synergistic effects of abietic acid combined with doxorubicin on apoptosis induction in a human colorectal cancer cell line
Source: Sci Rep. 2025 May 8;15:16102. doi: 10.1038/s41598-025-99616-2 (PMC12062260; doi:10.1038/s41598-025-99616-2)
Supplement: Supplementary file 7 — Supplementary Material 7 [file 41598_2025_99616_MOESM7_ESM.docx]

**Synergistic effects of abietic acid combined with doxorubicin on apoptosis induction in a human colorectal cancer cell line**


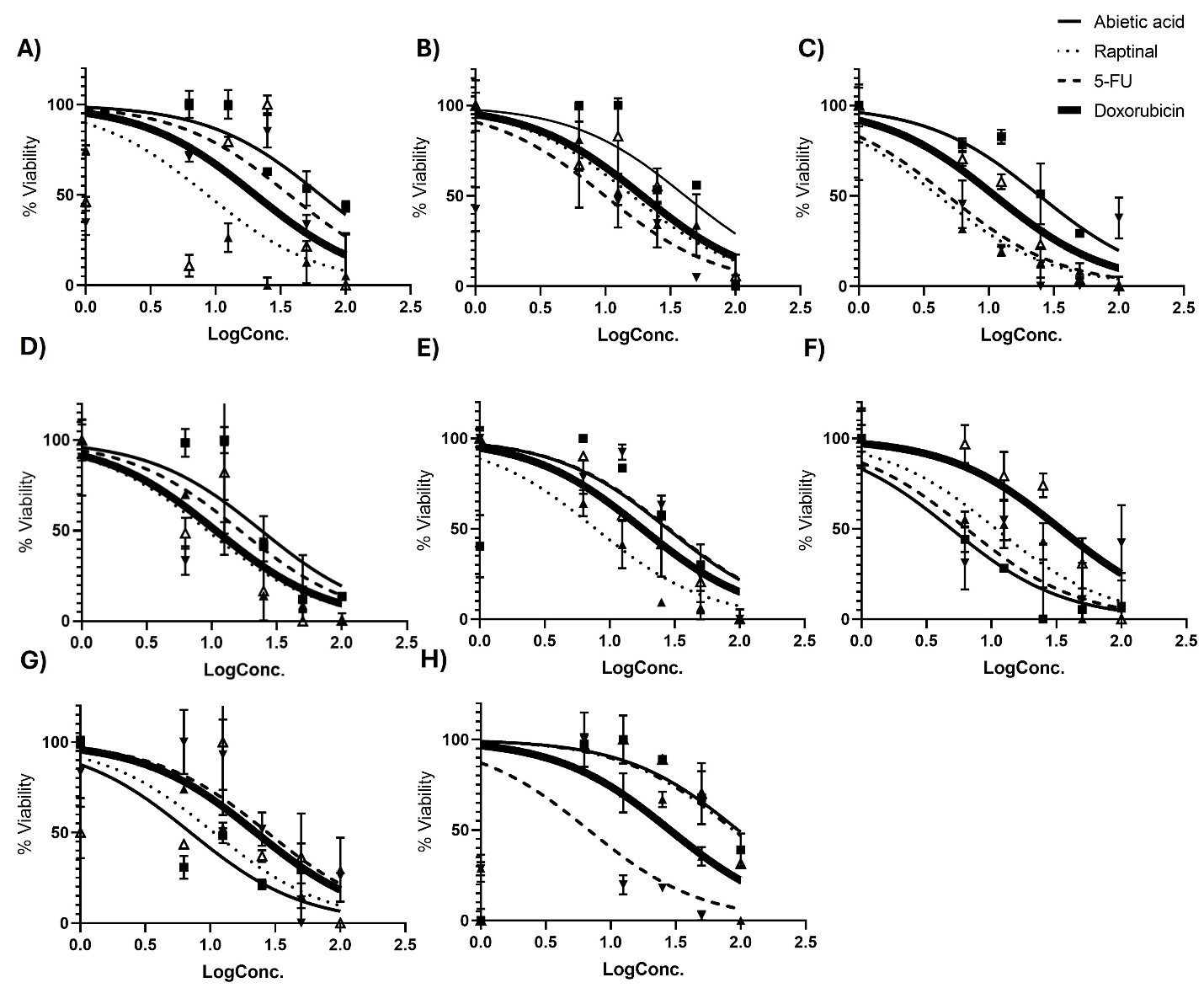


**Fig. 1S**: Cancer cell growth inhibition measured by MTT test. Sigmoidal dose-response curve of the investigated compounds (Abietic acid, Raptinal, 5-Fu and Doxorubicin) against seven cancer cell lines: A) Caco-2, B) DU-145, C) HCT-116, D) Hep-2, E) HepG-2, F) MDA-MB231, G) PC-3 and H) WI-38. "Y-axes represents the percentage of cell viability (normalized to control), and X-axes represent the values of log [concentration]". The presented figures are representation of three replicates of the experiment.
