# Supplementary material for: Synergistic effects of abietic acid combined with doxorubicin on apoptosis induction in a human colorectal cancer cell line
Source: Sci Rep. 2025 May 8;15:16102. doi: 10.1038/s41598-025-99616-2 (PMC12062260; doi:10.1038/s41598-025-99616-2)
Supplement: Supplementary file 8 — Supplementary Material 8 [file 41598_2025_99616_MOESM8_ESM.docx]

**Synergistic effects of abietic acid combined with doxorubicin on apoptosis induction in a human colorectal cancer cell line**


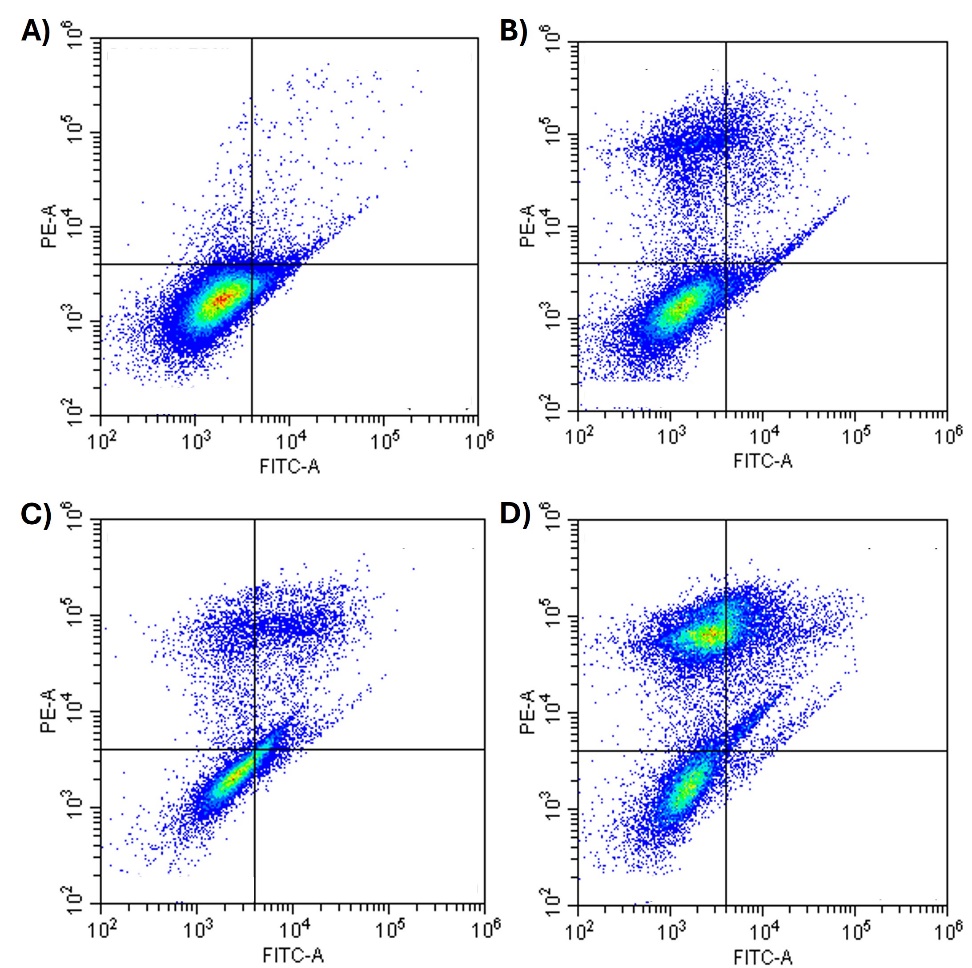


**Fig. 2S**: Apoptotic contour plots for HCT-116 cells treated with A) 0.1% DMSO act as negative control, B) IC_50_ dose of abietic acid, C) IC_50_ dose of doxorubicin and D) combined IC_50_ doses of abietic acid and doxorubicin combination measuring the percentage of viable, early apoptotic, late apoptotic, and necrotic cells by AV/PI assay using flow cytometry. The assay was performed after the treatment of HCT-116 (colon cancer) for 24h.
